# Natural variation in growth and leaf ion homeostasis in response to salinity stress in *Panicum hallii*

**DOI:** 10.3389/fpls.2022.1019169

**Published:** 2022-10-07

**Authors:** Taslima Haque, Govinal Badiger Bhaskara, Jun Yin, Jason Bonnette, Thomas E. Juenger

**Affiliations:** Department of Integrative Biology, University of Texas at Austin, Austin, TX, United States

**Keywords:** adaptation, ion transporter, natural variation, QTL, transcriptome remodeling, ion homeostasis

## Abstract

Soil salinity can negatively impact plants growth, development and fitness. Natural plant populations restricted to coastal environments may evolve in response to saline habitats and therefore provide insights into the process of salinity adaptation. We investigated the growth and physiological responses of coastal and inland populations of *Panicum hallii* to experimental salinity treatments. Coastal genotypes demonstrated less growth reduction and superior ion homeostasis compared to the inland genotypes in response to saline conditions, supporting a hypothesis of local adaptation. We identified several QTL associated with the plasticity of belowground biomass, leaf sodium and potassium content, and their ratio which underscores the genetic variation present in this species for salinity responses. Genome-wide transcriptome analysis in leaf and root tissue revealed tissue specific overexpression of genes including several cation transporters in the coastal genotype. These transporters mediate sodium ion compartmentalization and potassium ion retention and thus suggests that maintenance of ionic homeostasis of the coastal genotypes might be due to the regulation of these ion transporters. These findings contribute to our understanding of the genetics and molecular mechanisms of salinity adaptation in natural populations, and widens the scope for genetic manipulation of these candidate genes to design plants more resilient to climate change.

## 1 Introduction

Plants experience a host of complex environmental stresses such as soil salinity, drought, and high temperature in their natural habitats that can impact their growth, physiology, and ultimately their fitness. Plant populations may over time evolve adaptations that maintain fitness in the face of stressful local environmental conditions (Leimu and Fischer, 2008; Joe Hereford, 2009). Coastal habitats are often exposed to elevated soil salinity due to repeated inundation of soil by sea water, salt water spray, or saltwater intrusion to the fresh water table. In such habitats, salinity can act as a major driver of local adaptation (Lexer et al., 2003; Baxter et al., 2010; Busoms et al., 2015; Busoms et al., 2018). The underlying genetic basis and evolved molecular mechanisms for salinity adaptation have become of great interest due to the adverse effects of recent climate changes and risks of sea level rise. However, these studies are mostly limited to model plants and certain crops (Qiu et al., 2002; Ren et al., 2005; Davenport et al., 2007; Tiwari et al., 2016; Haque et al., 2020; Atieno et al., 2021) and are seldom conducted in natural populations (although see Lexer et al., 2003; Rus et al., 2006; Lowry et al., 2009; Baxter et al., 2010; Busoms et al., 2018). Studies in natural populations can facilitate the identification of novel genetic variation and our understanding of the underlying adaptive molecular mechanisms for salinity stress.

Plant adaptations to salinity can be categorized into three distinct tolerance mechanisms: osmotic stress tolerance, ion exclusion, and the tolerance of tissue to accumulated ions. (Munns and Tester, 2008; Roy et al., 2014). The osmotic tolerance mechanism is triggered before leaf Na^+^ accumulation by long distance signaling. In Arabidopsis, the hyperosmolarity-gated calcium channel (OSCA1) was identified as a putative hyperosmotic stress sensor that triggers cytosolic Ca^2+^ which subsequently induces stomatal closure (Zhang et al., 2022). In ion exclusion, Na^+^ transport processes in roots reduce the accumulation of toxic concentrations of ions within leaves and helps to maintain cellular ion homeostasis. Lastly, general tissue tolerance compartmentalizes excessive sodium ions into older leaf tissues and vacuoles. Cytosolic Na^+^ to K^+^ ratio can be a major determinant of salinity tolerance (Dvořak et al., 1994; Maathuis and Amtmann, 1999; Cuin et al., 2003; Colmer et al., 2006). The optimal cytosolic ratio of Na^+^ to K^+^ can be maintained by restricting Na^+^ accumulation in tissues, by retaining K^+^ inside the cell, or by both processes (Shabala and Cuin, 2008). Candidate genes responsible for ion homeostasis have been studied in many model plants and economically important crops. For instance, the high affinity potassium transporter (HKT) in Arabidopsis (HKT1;1), rice (HKT1;5) and wheat (HKT1;5) is involved in the retrieval of Na^+^ from xylem (Davenport et al., 2005; Ren et al., 2005; James et al., 2006; Davenport et al., 2007). Conversely, KT/HAK/KUP family potassium transporters have been reported to play a role in maintaining potassium homeostasis and help to confer salinity tolerance in rice (Obata et al., 2007; Chen et al., 2015; Shen et al., 2015).

Salinity tolerance has evolved independently among many different plant clades. For example, salt tolerant genotypes from natural populations of rice and rye grass maintain relatively low leaf Na^+^ concentration (Platten et al., 2013; Tang et al., 2013) and the natural hybrid species *Helianthus paradoxus* and coastal populations of *Mimulus guttatus* exhibit tissue tolerance (Karrenberg et al., 2006; Lowry et al., 2009). Natural populations of Arabidopsis exhibit osmotic adjustment which confers Na^+^ tolerance (Rus et al., 2006; Baxter et al., 2010). Pires et al. (2015) found no specific salinity tolerance mechanism was predominant among diverse rice natural populations and that tolerance mechanisms were not mutually exclusive. In the grass family salt tolerance evolves more frequently in C4 grass lineages, likely due to existing predisposition of traits that facilitate tolerance mechanisms (Bromham, 2014; Bromham and Bennett, 2014). Several empirical studies of salt tolerant C4 grasses reported superior ion homeostasis relative to more sensitive counterparts (Marcum and Murdoch, 1994; Fortmeier and Schubert, 1995; Pittaro et al., 2016). Hence, natural populations of C4 grasses which are native to saline habitats provide a valuable study system for adaptation to saline conditions.

Coastal population of the C4 perennial grass, *Panicum hallii*, experience higher soil salinity relative to inland populations that are typically found in seasonally water limited xeric habitats. *P. hallii* is widely distributed across the southwestern parts of the USA and northern Mexico and demonstrates substantial phenotypic and genetic divergence between the inland (*P. hallii* var. *hallii*) and coastal ecotypes (*P. hallii var filipes*) (Lowry et al., 2015; Lovell et al., 2018; Palacio-Mejía et al., 2021). Regulatory element evolution and gene expression divergence in response to drought has been detected in *P. hallii*, and underscores the significant contribution of drought stress as a driver of local adaptation in this species (Lovell et al., 2016; Lovell et al., 2018). However, the potential impact of soil salinity to adaptation in the coastal ecotype, and the likely presence of cross tolerance of inland population adaptation to more xeric habitats remains unexplored. In this study, we aim to i) evaluate ecotype and genotype specific phenotypic responses to experimental salinity treatment, ii) identify the genomic region contributing to salinity response, and iii) detect genotype specific transcriptome remodeling during salinity stress and prioritize candidate genes for such response.

## 2 Method

### 2.1 Response to salinity treatment in ecotypes of *P. hallii* (Experiment 1)

The first study was focused on exploring the broad pattern of responses to salinity by comparing natural accessions of *P. hallii* obtained from collections across Texas and New Mexico (Gould et al., 2018; Lovell et al., 2018; Palacio-Mejía et al., 2021). Here, seed from individual maternal lines were obtained from field collection and seed was bulked under greenhouse conditions in Austin Texas (**Supplementary Figure S1**). *P. hallii* is predominantly self-fertilized and exhibits high coefficients of inbreeding. As such, these accessions exhibit very low heterozygosity and highly inbred lines (mean inbreeding coefficient, F_IS_ = 0.895) (Lowry et al., 2015; Palacio-Mejía et al., 2021). To test for the response to salinity between the inland and coastal ecotypes, ten inbred genotypes from each ecotype were chosen for study in a factorial experiment (**Supplementary Table S1**). Replicates of this plant material were exposed to a realistic soil salinity stress treatment (20 genotypes × 2 treatment levels × 5 biological replicates = 200 plants) in a split-plot design. Blocks were randomly assigned to one level of treatment and all genotypes were nested in each block. First, seeds were treated at 65°C for three days to break dormancy, on the fourth day were sown on soil in individual pots (6:1:1 homogenous mixture of Promix: Turface: Profile) and seedlings were grown in a controlled growth chamber at 28/25°C for 12hr/12hr day/night cycle. Irrigation was implemented with bottom watering using a nutrient solution (see **Supplementary Method S1**, method section 1.2 for detailed methods) on alternative days. Pots were thinned on the 5th day after sowing (DAS) and only one healthy seedling was kept in each pot for the experiment. On that day, pots lacking a healthy seedling were discarded and any genotype which failed to germinate for at least 3 replicates in each genotype x treatment combination were removed from the experiment. This filtering resulted in 9 genotypes from the coastal group and 8 genotypes from the inland group (**Supplementary Table S1**). Plants designated for the salinity stress treatment received a concentrated NaCl solution (prepared in nutrient solution) progressively (83 mM, 167 mM and 250 mM) starting at 31^st^ DAS while the control group received only nutrient solution. This treatment was continued for six days (nutrient/salinity solution was applied on each alternative day at 31^st^, 33^th^, and 35^th^ DAS) until leaf relative water content, aboveground biomass, and belowground biomass were measured on 37^th^ DAS (**Supplementary Method S1** for detailed methods). A salt tolerance index was derived as the ratio of mean trait values for salinity treated plants to control plants for a given genotype for aboveground and belowground biomass. During the course of salinity treatment, the soil salinity of each pot was measured at a depth of ~1 inch using a field electrical conductivity (EC) meter (Spectrum Technologies) after 2 h of treatment application. On the 35^th^ DAS mean soil salinity for treatment pots was 12.9 deciSiemens/m (dS/m) which is equivalent to ~130 mM NaCl solution and often considered moderate salinity stress for many species (Munns and Tester, 2008; Isayenkov and Maathuis, 2019).

### 2.2 Response to salinity of two genotype (Experiment 2)

Detailed physiological and growth response of plants to the salinity treatment were tested on two genotypes of *P. hallii* [one representative genotype of the inland *hallii* ecotype (HAL2) and one representative genotype of the coastal *filipes* ecotype (FIL2)] with 12 replicates in each genotype x treatment combination (2 treatment levels x 2 genotypes x 12 biological replicates = 48 plants). These plants were established in a split-plot design. Blocks were randomly assigned to one level of treatment and genotypes were nested in each block. Plant growth condition and treatment application were carried out in the same manner described for the population study except this time we applied a different concentration of NaCl solution progressively (100 mM, 200 mM and 300 mM). Based on our results from Experiment 1, a moderately higher level of salinity stress was chosen to test for the response at the genotype level. On 37^th^ DAS aboveground fresh biomass, belowground fresh biomass, the ratio of fresh biomass (aboveground/belowground), leaf relative water content, leaf water potential, leaf sodium content (Na^+^), leaf potassium content (K^+^), and the ratio (Na^+^/K^+^) were measured. Leaf water potential was measured by using a Scholander-type pressure chamber (PMS Instruments Company, Albany, OR). Detailed protocols for measuring leaf osmotic potential, leaf relative water content, and ion contents were provided in the **Supplementary Method S1**.

### 2.3 Response to salinity of *P. hallii* RIL population (Experiment 3)

To further study the genetic basis of salinity responses in *P. hallii*, we implemented a QTL mapping study using recombinant inbred lines (RILs) from a cross between *P. hallii* var. FIL2 and *P. hallii* var. HAL2. This mapping population consists of 380 RILs developed by single seed descent (Khasanova et al., 2019). This QTL mapping experiment was based on a randomized block design and allocated 3 replicates of each RIL to a control or salinity treatment. Given the size and scope of the experiment, the experiment was blocked in time by splitting the experiment into 3 cohorts with each cohort containing a full set of the mapping population plus 10 replicates of each parental genotype (each cohort comprised of 380 RILs + 2 parents x 10 replicates] x 2 treatment levels = 800 plants; 3 cohorts x 800 plants = 2400 plants total). Plant growth and treatment application was carried out as described for the population salinity response experiment except this experiment was carried out in a controlled greenhouse at the University of Texas at Austin in spring 2019 with natural light providing long day conditions (~14h/10 h day/night). Each day temperature was recorded at one-minute intervals and the mean recorded temperature was 28/25°C during day/night. Plants were harvested at 37^th^ DAS and aboveground biomass (AGB), belowground biomass (BGB), the ratio of aboveground to belowground biomass (RB), leaf relative water content (RWC), Na^+^, K^+^, and the ratio (Na^+^/K^+^) were measured (see **Supplementary Method S1** detailed methods). Given the cost and labor associated with obtaining sodium and potassium ion content of our large experiment, only the salinity treated samples of the first cohort were considered for these two traits.

### 2.4 Statistical analysis for phenotypes

Linear mixed models were fitted using the lmer package from R software to analyze the factorial experimental design (Bates et al., 2015). The ecotype, treatment and the interaction of ecotype x treatment were considered as fixed effects and the effect of block, the effect of block nested in treatment and the effect of genotypes nested in ecotypes were considered as random effects for the traits measured in experiment 1. For traits measured in experiment 2 we considered the effect of genotype (HAL2 versus FIL2 genotypes), treatment and the interaction as fixed effects and experimental block, and the nested effect of block in treatment as random effects. While analyzing traits for parental genotypes in experiment 3, a model was fitted in which treatment, genotypes and the interaction were incorporated as fixed effects while cohort was considered as a random effect. The significance of fixed effects was tested by F-tests using Satterthwaite’s method to obtain p-value and the degree of freedom.

### 2.5 QTL mapping

A genetic linkage map composed of 901 markers evenly distributed across the nine *P. hallii* chromosomes was used for QTL mapping analysis. Each marker represented a window of 50 high quality SNPs called from the re-sequencing data of RIL individuals (NCBI SRA archive Umbrella project PRJNA701489). A detailed description of the linkage map construction for this RIL mapping population can be found on Dryad (https://doi.org/10.5061/dryad.73n5tb2w8). In this RIL mapping experiment all the traits except Na^+^, K^+^ and Na^+^/K^+^ were measured in both control and salinity treatment conditions. As such, this experimental design can be considered a two factorial design and our aim was to identify the main effect of inland versus coastal alleles (G) on phenotypic variation and the interactive effect of coastal/inland allele with treatment (GxT) on phenotypic variation. This analysis strategy relied on a reaction norm perspective. Here, the QTL mapping analysis was split into a portion based on the simple additive effect of QTL averaged across the control and salinity treatment by taking the average of replicates from the two treatment levels. This analysis will detect QTL that are robust to the environments and that have constitutive effects. To study the QTL that show QTL x salinity treatment effects, the difference among the replicates across the treatment was also studied (Lowry et al., 2013). Prior to running QTL models the normality of each trait was tested and transformed with a Box-Cox transformation when required (Box and Cox, 1964). The “scanone” function in the R/qtl (Broman et al., 2003) package was used to detect QTL for constitutive and responsive traits by using the following models:

QTL_Constitutive_: Y_mean_ (mean of control and stress for each RIL) = µ + QTL + cohort (if applicable) + QTL x cohort (if applicable) *+ error*
QTL_Responsive_: Y_Diff_ (Difference from Stress to Control) = µ + QTL + cohort (if applicable) + QTL x cohort (if applicable) *+ error*
The following QTL model was implemented for the traits which were measure only on salinity treated plants (Na^+^, K^+^ and Na^+^/K^+^):
QTL_Ionic_: Y_Treat_ (Trait measure in treatment condition) = µ + QTL *+ error*

To detect potential epistatic interactions the “scantwo” function was implemented. Permutation (n=1000) for each trait was performed with stratification by cohort to obtain the null distribution for main and epistatic interaction effects. The stepwise QTL function was used to complete a forward-backward search by adding/dropping QTL effects and their epistatic effect. The threshold value for Type I error was set as 0.1 for all the traits and QTL intervals were calculated with a threshold of 1.5 LOD drop from the peak. Estimated QTL effects were obtained by the “qtlStats” function from the qtlTools package (Lovell, 2018) with the final QTL model for a given trait. Detailed methods for QTL mapping is provided in **Supplementary Method S1**, section 1.10. Gene models residing in a given QTL confidence interval were considered as candidate genes for that specific QTL and GO enrichment analysis was carried out as mentioned in **Supplementary Method S1**, section 1.9. All gene models which were not residing in QTL confidence intervals were considered as the genomic background for enrichment analysis.

### 2.6 TAGSeq library construction and identification of differentially expressed genes

To characterize the transcriptome response to salinity a RNA-sequencing strategy was implemented. To do so, ~8 replicates of each genotype (HAL2 or FIL2) x treatment combination from the mature plant experiment described in section 2.1 were randomly selected for further characterization. The first emerging leaf and the total root systems of plants were sampled on 37^th^ DAS around 10 AM to study global gene expression. 3′ TAGSeq libraries were constructed as described by Weng et al. (2019) and sequenced with 1x150 bp single-end reads on HiSeq 2500 (Illumine, San Diego, CA, USA). Raw reads were quality filtered, mapped to the *P. hallii* var. *hallii* (HAL2) reference genome (v2.0), filtered for mapping quality, and expression counts were generated using the v2.1 (https://phytozome-next.jgi.doe.gov/info/PhalliiHAL_v2_1) gene models of the mentioned reference genome. Raw reads can be found in NCBI Bioproject PRJNA853054. Detailed method for library construction and count matrix generation can be found in **Supplementary Method S1**, section 1.7 and sequencing statistics are provided in Supporting **Table S8**. The DESeq2 package (Love et al., 2014) in R (R Core Team, 2021) was used to test for differentially expressed genes (DEG) at genotype (G), treatment (T) and genotype x treatment (GxT) levels. Detailed methods for this analysis are described in the supplementary method section. In brief, libraries were normalized for their size and dispersion was estimated using the fitted dispersion-mean relationship. DEG were identified by running gene-wise likelihood ratio tests (LRT) and compared the full model with interaction to corresponding reduced models. The Benjamini & Hochberg method (Benjamini and Hochberg, 1995) of False Discovery Rate (FDR) was used to account for multiple testing and significance was determined by an adjusted p-value <0.05. Gene Ontology (GO) enrichment was tested for different sets of DEG using the topGo package (Adrian and Rahnenfuhrer, 2021) against detected expressed genes for a given tissue type as background. GO terms with adjusted p-value <0.1 were considered significant. To evaluate the global plasticity for transcriptomic response to salinity treatment, Discriminant analysis of Principal Components (DAPC) was implemented on normalized (by library size) and transformed count data with variance stabilizing transformation (VST) using the fitted dispersion-mean. DAPC is a multivariate analysis method designed to identify clusters and their separation in multivariate space. It synthesizes linear discriminatory functions in such a way that maximizes between group variance while minimizing within group variance. DAPC was carried out on the normalized data with four predefined groups (genotype x treatment combinations) using the adegenet package (Jombart et al., 2010). Subsequently, global transcriptome plasticity in response to the salinity treatment was measured as the Euclidean distance (of first two linear discriminatory axes) from each biological replicate at a given genotype in salinity treatment group to the mean of the control group for that given genotype.

### 2.7 Codes availability

All code and scripts for the analyses and plotting can be found in https://github.com/tahia/NatVariation_salinity_adaptation_Phallii.

## 3 Result

### 3.1 Phenotypic response to salinity treatment in *P. hallii*

**Figure 1:**
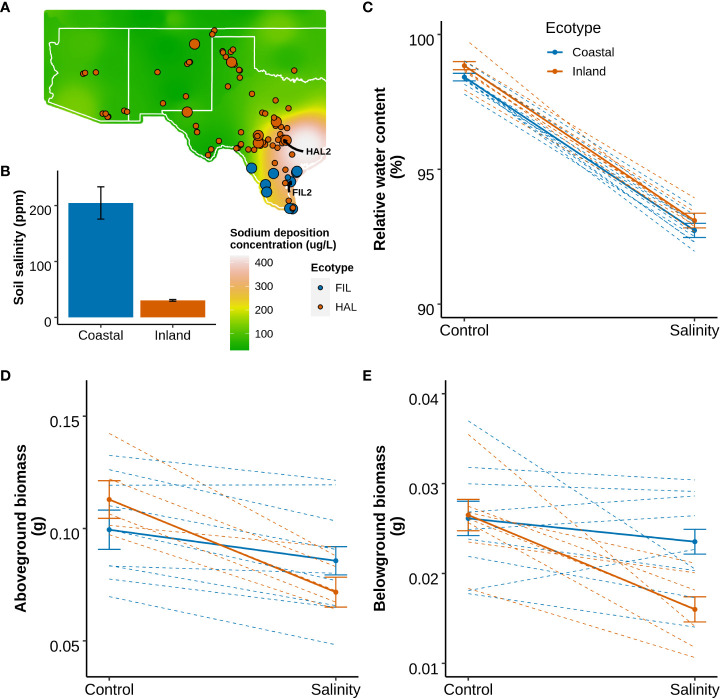
Geographical distribution of *P. hallii* from natural collections and the phenotypic response to salinity of selected inland and coastal accessions. **(A)** Geographical distribution of *P. hallii* in which filled points represent the collection location of accessions while color represents ecotypic variation. Raster plot represents annual soil sodium deposition collected from National Atmospheric deposition program for the year 2018 (https://gaftp.epa.gov/castnet/tdep/2021_01_grids/). Points with larger radius represent the selected inland and coastal accessions that have been tested in our salinity response experiment. **(B)** Bar plot of sodium concentration measured from the top soil of several coastal and inland habitats. **(C–E)** Reaction norm plots at two different treatment levels (Control and Salinity stress) for leaf relative water content, aboveground biomass, and belowground biomass respectively. The solid and dotted line represents ecotype specific and genotype specific group means respectively.

The species range of *P. hallii* spans from the saline rich coastal habitats along the Gulf Coast of Texas to the arid Chihuahuan desert in the southwest. The edaphic conditions vary considerably across this distribution, and exhibit a strong soil sodium deposition gradient (**Figure 1A**). Approximately 6.6-fold higher topsoil sodium concentration (p-value=0.01) was detected in the coastal habitats compared to the inland habitats (**Figure 1B**; **Supplementary Method S1** section 1.1). To evaluate the differential response of coastal and inland ecotypes, ten genotypes from each ecotype were selected, applied a soil salinity stress as a sodium chloride solution supplement, and leaf relative water content, and aboveground and belowground biomass (Method section 2.1: Experiment 1). Under a model of local adaptation, we hypothesized that the coastal ecotype might perform better than the inland ecotype under salinity stress. A significant effect of ecotype-by-treatment interaction was detected for both aboveground (p-value=0.04) and belowground biomass (p-value=0.02), whereas only a significant effect of treatment was detected for relative water content (**Figure 1** and **Supplementary Table S2**). The inland ecotype had a significantly lower salt tolerance index (the ratio of mean trait value for salinity treated plants to control plants) compared to the coastal ecotype for both the biomass traits (p-value < 0.05 for one-way ANOVA). The reduction of growth in response to salinity was 24% and 32% higher in the inland ecotype compare to the coastal ecotype for aboveground and belowground biomass respectively. This result implies improved capacity of the coastal ecotype for growth maintenance when exposed to salinity stress.

### 3.2 Phenotypic response to salinity treatment in of *P. hallii* inland and coastal genotypes

**Figure 2:**
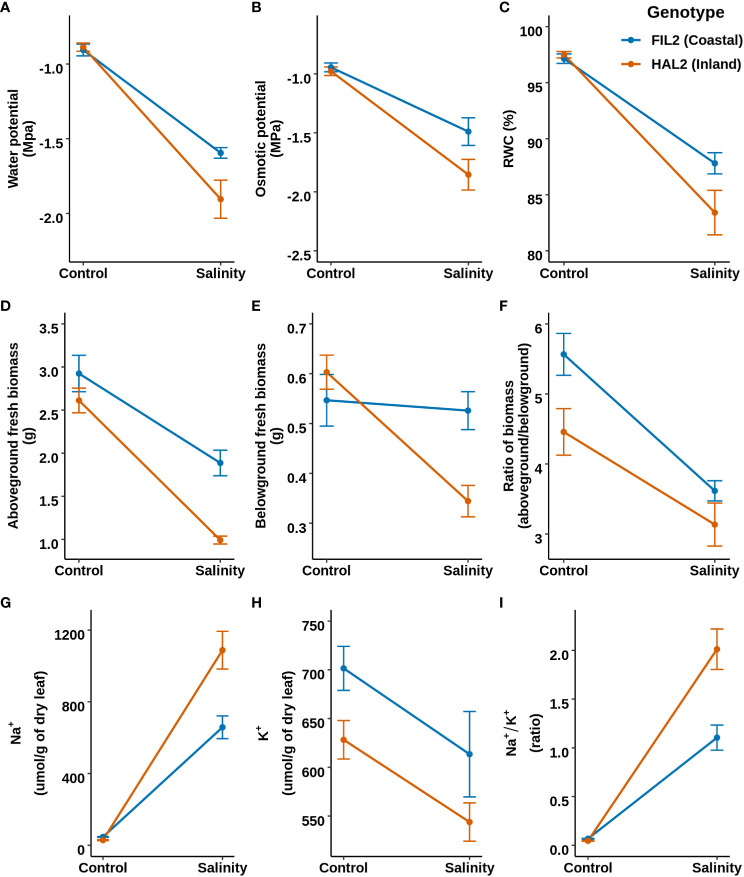
Response of mature plants to salinity based on representative genotypes of coastal (FIL2) and inland (HAL2) ecotypes. Top, middle, and bottom panels represent the reaction norm plots for leaf water status, plant growth, and leaf sodium and potassium ion homeostasis related traits, respectively. **(A–C)** Water potential, Osmotic potential, and Relative water content respectively. **(D–F)** represents the reaction norm of Aboveground fresh biomass, Belowground fresh Biomass, and their ratio (aboveground/belowground). **(G–I)** represents leaf sodium content (Na^+^), Leaf potassium content (K^+^), and the ratio of leaf sodium to potassium content (Na^+^/K^+^).

To evaluate the response to salinity in greater detail, one representative genotype of each ecotype (the inland ecotype: HAL2 and the coastal ecotype: FIL2; the genome reference genotypes for *P. hallii*) were selected and a salinity stress was applied (Detailed in **Supplementary Method**: Experiment 2). We hypothesized that the coastal genotype might perform more efficiently compared to the inland genotype at additional physiological and growth parameters (section 3.1) related to salt tolerance. Among the traits related to leaf water status and osmolarity, leaf water potential demonstrated significant genotype-by-treatment interaction (GxT) whereas osmotic potential and relative water content exhibited significant treatment effects (T) only (p-value < 0.05; **Supplementary Table S3** for detailed test statistics) (**Figure 2**). The coastal genotype maintained a 16% less negative water potential (lower water stress, p-value= 0.02) compared to the inland genotype, without detectable genotype specific changes in relative water content under salinity treatment compared to control condition. Among growth related traits, aboveground and belowground fresh biomass showed significant GxT, the inland genotype exhibited a 43% decrease of belowground fresh biomass in response to salinity stress (p-value=1.4e^-05^) compared to control, while the coastal genotype demonstrated no significantly detectable reduction. Similarly, Na^+^ and Na^+^/K^+^ showed significant GxT and the coastal genotype exhibited significantly lower Na^+^ (40% less, p-value=0.002) and Na^+^/K^+^ (45% less, p-value=0.001) under salinity treatment compared to the inland genotype (**Figure 2**). Overall, in response to salinity stress the coastal genotype maintained higher shoot and root growth, less stressful water status, and better ion homeostasis compared to the inland genotype.

### 3.3 Detection of QTL for salinity response in RIL mapping population

To identify the genomic regions underlying the differential response to salinity stress, quantitative trait loci (QTL) mapping was carried out using the RIL mapping population from HAL2 and FIL2 parents. In congruence to our earlier findings (see section 3.2), belowground biomass, Na^+^, and Na^+^/K^+^ showed significant GxT for parents (p-value < 0.05, **Supplementary Table S4**: Summary statistics). On the other hand, aboveground biomass exhibited significant additive effect (p-value < 0.05) of genotype and treatment but no interaction.

**Figure 3:**
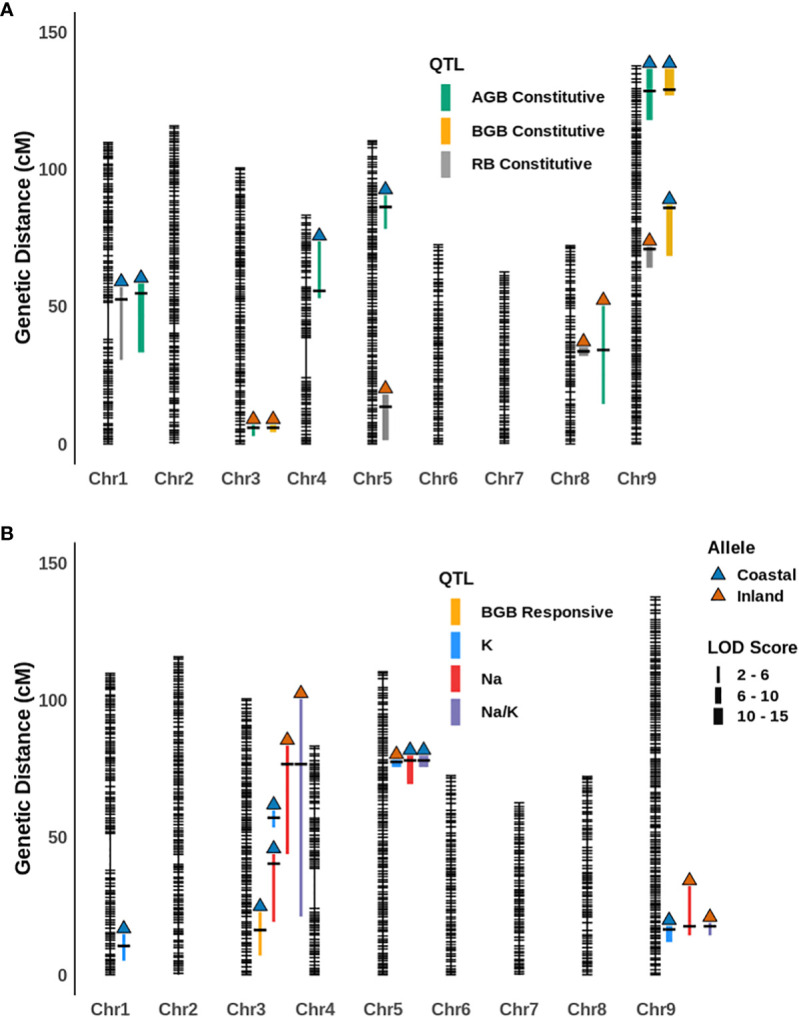
The position of **(A)** constitutive and **(B)** responsive category and ionic QTL for various traits on the genetic map of the *P. hallii* RIL population. Vertical color filled bar indicates QTL intervals estimated by 1.5 LOD drop from the peak. The width of the bar represents the magnitude of the LOD score for the given QTL and different colors represents different traits. The color of upward arrow indicates the genotype for alleles that have a positive effect for a given trait.

In this QTL analysis framework, the overall mean and difference of genotypic values between treatment levels for a given trait were defined as either ‘constitutive’ or ‘responsive” respectively for our measures phenotypes (**Supplementary Table S5**). The narrow-sense heritability (h^2^) for traits on the mean, difference and ion homeostasis related traits (in the salinity treatment) ranged from 0-21%, 0-5% and 24-36% respectively (**Supplementary Table S6**). Very low heritability was observed for relative water content for both constitutive (0%) and responsive categories (0.04%). In the constitutive category, six, three, and four constitutive QTL were detected for aboveground biomass, belowground biomass, and the ratio respectively. These QTL explain 7.88%, 28.54% and 29.52% variance of the respective traits based on their final multiple QTL models (**Figure 3**; **Table 1**; **Supplementary Table S7**; **Supplementary Figure S2**). The coastal alleles increased trait values (positive effect) for the majority of QTL detected for aboveground and belowground biomass, whereas for the ratio (aboveground/below ground) inland alleles increased the ratio. Among these constitutive QTL, qAGB-5@85.8 (read as qTRAIT-Chromosome-Position), qBGB-8@33.1, and qBGB-3@5.4 shared the three QTL cluster intervals reported for shoot biomass, root diameter and specific root area respectively by Khasanova et al. (2019) from the same RIL mapping population. In the responsive category a single QTL, qAGB-5@85.8 was detected for belowground biomass with an interval of ~ 15 cM. This QTL explained 1.38% of variance of the response difference in belowground biomass and the coastal allele had a positive effect (less reduction of belowground biomass in response to salinity). Four QTL were detected for each leaf potassium and leaf sodium content models. For the ratio of leaf sodium to potassium content three QTLs were identified. Subsequently, multiple QTL models explained 29.1%, 21.4%, and 25% of variance for K^+^, Na^+^ and Na^+^/K^+^. Among the four QTL detected for K^+^, three of these (qK-1@10, qK-3@56.7 and qK-9@17.2) had positive effect from coastal alleles; however, the fourth QTL (qK-5@77) had the largest and positive effect from the inland allele. In addition, an epistatic interaction between qK-1@10 and qK-9@17.2 was detected with RILs that possess either or both coastal alleles at these loci maintaining higher leaf potassium than RILs with both inland alleles (**Supplementary Figure S3**). Out of the four QTL detected for Na^+^, for two QTL (qNa-3@76.2 and qNa-9@17.2) the inland alleles increased Na^+^ while for the other two (qNa-3@40 and qNa-5@77.5) the coastal alleles elevated the trait. Noticeably, all three QTL detected for the ratio of leaf sodium to potassium content (qNa/K-3@76.2, qNa/K-5@77.5, and qNa/K-9@17.2) overlap with the intervals of detected QTL for Na^+^ and K^+^ individually resulting in three ionic QTL clusters. Since Na^+^ and K^+^ demonstrated a weak negative correlation (Pearson correlation coefficient = -0.29) in this mapping population, these QTL clusters imply a plausible association of these loci with ionic balance. Altogether, several genetic loci were identified that were associated with the response difference of belowground biomass and the ionic homeostasis related traits during salinity stress. Generally, the coastal genotype contributed alleles towards a more resilient response. However, the detection of several QTL with inland alleles as positive contributors for ionic homeostasis suggests that the inland genotype may also possess genetic regulators of ionic homeostasis.

**Table 1:** List of QTL for Constitutive, Responsive and Treatment Categories.

QTL Name	Category	Trait	Chromosome	Peak (cM)	Interval (cM)	LOD	Positive Allele
**qK-1@10.0**	Ionic	K^+^	1	10.0	5.1-14.1	4.95	FIL2
**qK-3@56.7**	Ionic	K^+^	3	56.7	53.7-59.9	5.39	FIL2
**qK-5@77.0**	Ionic	K^+^	5	77.0	75.6-78.3	12.28	HAL2
**qK-9@17.2**	Ionic	K^+^	9	17.2	11.9-17.9	6.21	FIL2
**qNa-3@40**	Ionic	Na^+^	3	40.0	19.2-43.9	3.08	FIL2
**qNa-3@76.2**	Ionic	Na^+^	3	76.2	43.9-83.5	2.8	HAL2
**qNa-5@77.5**	Ionic	Na^+^	5	77.5	69.4-79.8	9.06	FIL2
**qNa-9@17.2**	Ionic	Na^+^	9	17.2	14.3-32.3	3.65	HAL2
**qNa/K-3@76.2**	Ionic	Na^+^/K^+^	3	76.2	21.2-100.5	2.4	HAL2
**qNa/K-5@77.5**	Ionic	Na^+^/K^+^	5	77.5	75.6-79.8	14.11	FIL2
**qNa/K-9@17.2**	Ionic	Na^+^/K^+^	9	17.2	14.3-19	4.36	HAL2
**qBGB-3@15.8**	Responsive	BGB	3	15.8	7.0-22.9	2.8	FIL2
**qAGB-1@54.3**	Constitutive	AGB	1	54.3	33.3-58.4	6.17	FIL2
**qAGB-3@5.4**	Constitutive	AGB	3	5.4	2.9-7	5.03	HAL2
**qAGB-4@55.2**	Constitutive	AGB	4	55.2	53.0-73.7	3.64	FIL2
**qAGB-5@85.8**	Constitutive	AGB	5	85.8	78.3-90.6	2.6	FIL2
**qAGB-8@33.7**	Constitutive	AGB	8	33.7	14.5-50.3	1.97	HAL2
**qAGB-9@128.0**	Constitutive	AGB	9	128.0	117.9-136.6	7.61	FIL2
**qBGB-3@5.4**	Constitutive	BGB	3	5.4	4.3-7	8.09	HAL2
**qBGB-9@85.4**	Constitutive	BGB	9	85.5	68.4-87.1	6.88	FIL2
**qBGB-09@128.5**	Constitutive	BGB	9	128.5	126.9-136.6	13.15	FIL2
**qRB-1@52.1**	Constitutive	RB	1	52.1	30.6-57.1	4.67	FIL2
**qRB-5@13.0**	Constitutive	RB	5	13.0	1.3-18.1	8.39	HAL2
**qRB-8@33.2**	Constitutive	RB	8	33.2	32.0-35.3	11.25	HAL2
**qRB-9@70.5**	Constitutive	RB	9	70.5	64.2-71.9	8.67	HAL2

### 3.4 Candidate genes within QTL intervals and their functional enrichment

To understand the molecular function of genes residing in ‘responsive’ or ‘ionic’ QTL intervals (average length of QTL interval ~18 cM and ~715 candidate genes/QTL interval) during salinity response, the enrichment of specific Gene Ontology (GO) terms in a given QTL interval was tested (Method section 2.4). GO terms such as response to oxidative stress, antioxidant activity and anatomical structure development were significantly enriched (adjusted p-value < 0.1) for qBGB-3@15.8. This result suggests that the presence of potential candidate genes associated with stress responses may contribute to the maintenance of belowground growth in saline conditions. Intervals for qNa-5@77.5 and qNa/K-5@77.5 were found to be enriched with response to oxidative stress and antioxidant activity while intervals for qK-9@17.2, qNa-9@17.2, and qNa/K-9@17.2 were enriched with oxidoreductase, transferase and phosphorelay sensor kinase activity. Since these QTL resided in two major QTL clusters it was not surprising that these intervals would be enriched with similar GO terms. Apart for these two treatment QTL clusters, the qNa-3@40 interval was enriched with cell redox homeostasis and chemical homeostasis terms. In the candidate gene lists for the majority of ion content QTL, we noticed the presence of three *a priori* gene families which have been well studied and reported to play key roles in salinity response: the *HKT* gene family (Davenport et al., 2005; Ren et al., 2005; Davenport et al., 2007), genes in the *SOS* pathway (Qiu et al., 2002; Møller et al., 2009; Ji et al., 2013) and the *KT/KUP/HAK* family of potassium transporter genes (Obata et al., 2007; Chen et al., 2015; Shen et al., 2015). The frequency of these *a priori* gene families (*HKT*, genes in *SOS* pathway, or *KT/KUP/HAK* family potassium transporter genes) in a given QTL interval was compared to random genomic background using a permutation test (**Supplementary Method S1**, section 1.11). Four QTL intervals, qK-1@10, qK-5@77, qNa-5@77.5 and qNa/K-5@77 were enriched with the HKT gene family (p-value < 0.05). Overall, the ion homeostasis QTL intervals were enriched with genes associated with oxidoreductase and chemical homeostasis activity and significantly enriched with *HKT* genes. These functional categories could be the likely candidates conferring salinity tolerance in *P. hallii*.

### 3.5 Global patterns of gene expression in response to salinity

**Figure 4:**
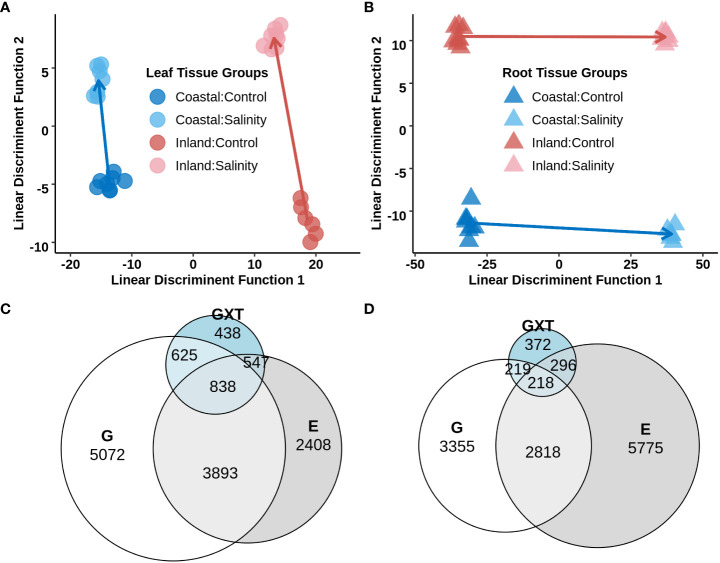
Differential gene expression in leaf and root tissues of the inland (HAL2) and coastal (FIL2) genotype of *P. hallii* in response to salinity stress. Top panel: **(A, B)** Discriminant Analysis of Principal Components (DAPC) on the normalized expression of genes from leaf (left, **A**) and root (right, **B**) tissues. Each point represents a single TAGSeq library and axes represents the first two linear discriminant functions. Symbols representing tissue types and genotype x treatment combinations are marked with different filled colors. Bottom pane: **(C, D)** Venn diagram for significant DEGs detected for Genotype (G), Treatment (T), and the interaction of Genotype with Treatment (GxT) as fixed effect for leaf and root tissue respectively.

To study transcriptional reprogramming during salinity stress, the first fully expanded leaf and the total root systems from experiment 2 were sampled (section 3.2; **Supplementary Table S8**) to obtain genome-wide gene expression profiles using a 3′- TAGSeq protocol (Weng et al., 2019). Among the 33,263 annotated gene models, 17,188 and 20,903 genes were detected in leaf and root tissue respectively (each with a mean of >4 count across the libraries for a specific tissue) and 16,225 genes which were shared in both tissues. A great deal of expression divergence was observed between libraries from leaf and root tissues (**Supplementary Figure S4**) and therefore differential gene expression analyses were conducted separately for each tissue type. To explore the global variation of gene expression within each tissue, the normalized transcript count data from a given tissue was used and Discriminant Analysis of Principal Components (DAPC) was applied with predefined groups corresponding to each genotype x treatment combination. Leaf tissue exhibited a strong signal of genotype on the first linear discriminatory function (**Figure 4A**) of the transcriptome. In contrast, the first discriminatory function separated the two treatment levels (**Figure 4B**) for root tissue. The inland genotype exhibited stronger plastic transcriptome responses to the salinity treatment compared to the coastal genotype in both leaf and root tissues (p-value < 0.05 for one-way ANOVA), but the difference of plasticity was much higher in leaf tissue. The elevated inland plasticity in leaf tissue compared to the coastal genotype could be due to ion exclusion that restricted transportation of Na^+^ from root to leaf tissue in the coastal genotype.

### 3.6 Differentially expressed genes in leaf tissue

To investigate the contribution of genotype and salinity treatment on gene expression profiles the expression of each detected gene was analyzed using a generalized linear model including Genotype (G), Treatment (T) and Genotype x Treatment interaction (GxT). Detected differentially expressed genes (DEGs) were categorized in four groups based on their fixed effects; DEG that had significant i) solitary Genotype effect (G), ii) solitary Treatment effect (T), iii) both Genotype and Treatment additive effects (G+T) and iv) an interaction of Genotype x Treatment (GxT). In leaf tissue 5072, 2408, 3893, and 2448 genes were identified for G, T, G+T and GxT categories respectively (**Figure 4** and **Supplementary Table S9**). Our specific interest was in the molecular function of DEGs which were upregulated during salinity treatment in the T, G+T or GxT categories and tested for GO enrichments separately in each of these groups. DEGs in these categories were enriched with various GO terms related to transmembrane signaling receptor activity and transmembrane transporter activity (nominal p-value <0.05, **Supplementary Table S10**). DEGs which were upregulated in G+T category were also enriched with oxidoreductase activity (p-value <0.05). Overall, several thousand genes were detected which demonstrated GxT interaction for their expression in leaf tissue, including the presence of various monovalent cation transporters and potassium channels. These results strengthen the hypothesis that inland and coastal expression divergence might impact ionic homeostasis during salinity stress.

### 3.7 Differentially expressed genes in root tissue

For root tissue, 3,355 genes were detected that exhibited significant expression variation only for genotype (G category), 5,775 genes showed significant variation only for treatment (T category), 2,818 genes displayed both genotype and treatment specific additive effects (G+T category) and 1,105 genes demonstrated significant genotype specific response to salinity (GxT category) (**Figure 4** and **Supplementary Table S11**). DEGs which were upregulated in the T category were enriched with transferase activity whereas upregulated DEGs in the G+T category were enriched with GO terms such as oxidoreductive activity and antioxidant activity (nominal p-value < 0.05, **Supplementary Table S12**). On the other hand, DEGs in GxT category were enriched with ion transport, drug transport, response to drug, and antiporter activity along with GOs such as oxidoreductive and antioxidant activity also found in upregulated G+T DEGs. In general, several cation transporters were detected in the GxT category in a fashion similar to the detected pattern of expression in leaf.

### 3.8 Differentially expressed ion transporters across leaf and root tissues in response to salinity

**Figure 5:**
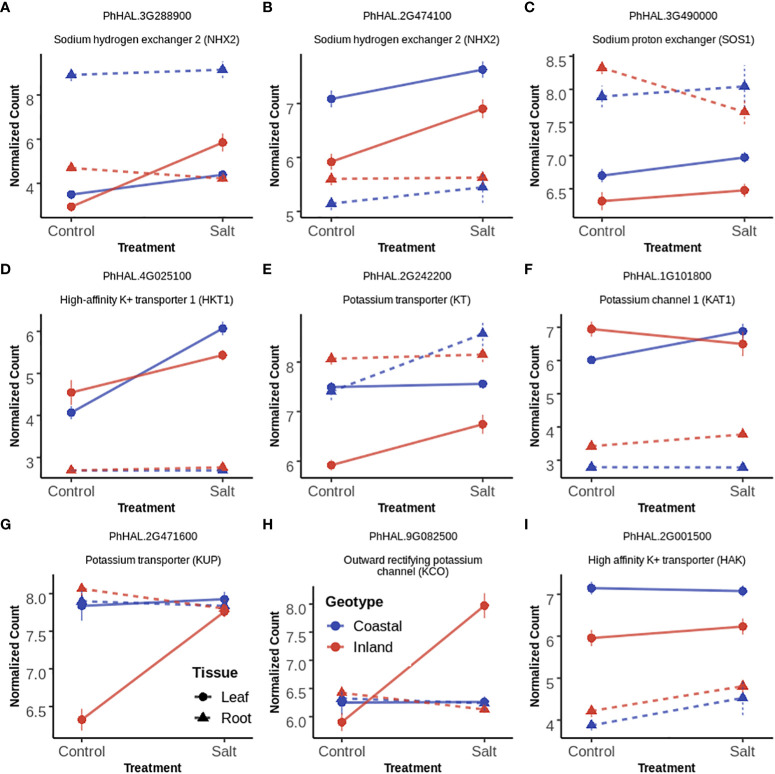
Gene expression profile of selected ion transporters (**A–I** panels) in leaf and root tissues with Treatment (T), both Genotype and Treatment (G+T) and Genotype by Treatment (GxT) interaction effects. In each panel, each point and error bar represent the mean and 1 SE of normalized gene expression of a selected gene for a given genotype, treatment and tissue level. Normalization was carried out by Variance Stabilizing Transformation (VST). Lines represent the change of gene expression for a given tissue and genotype in response to salinity while the color of lines represent the genotypes. Types of line represents tissue type (solid=leaf tissue; dotted=root tissue).

**Figure 6:**
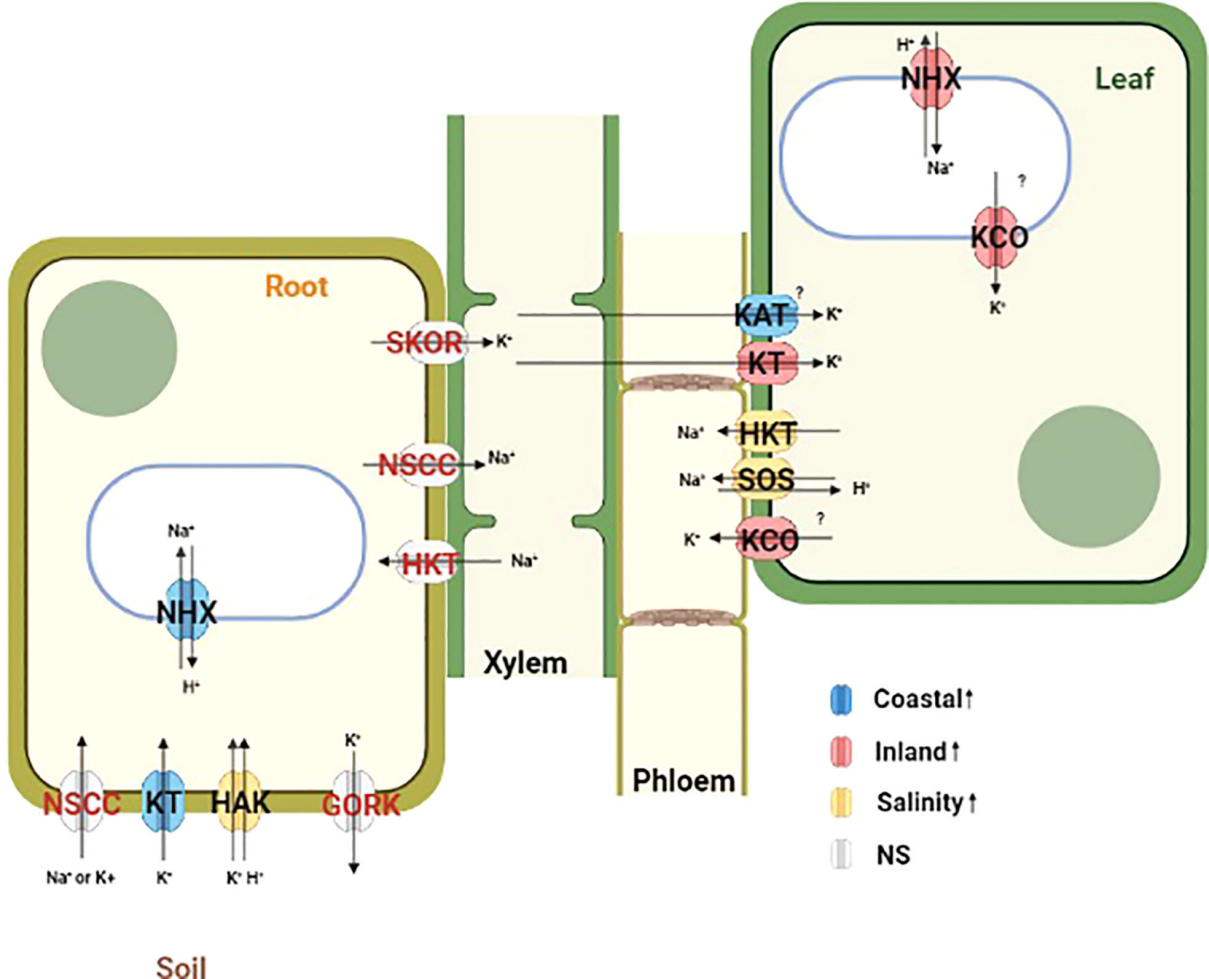
Schematic diagram representing the localization and function of *a priori* ion transporters and vacuolar ATPases which plays important role in maintaining ion homeostasis during salinity stress. Differentially expressed genes from this study are mapped in this diagram based on the functional annotation of *Panicum hallii* gene models. Ion transporters are color coded by their expression patterns (Blue=Coastal genotype specific overexpression, Red=Inland genotype specific overexpression, Yellow=Overexpression during salinity treatment irrespective of genotype and Gray=No significant detectable overexpression).

The analysis of differentially expressed gene demonstrated enrichment of transmembrane and ion transporters in T, G+T and GxT category genes for leaf and root tissue separately. Cation transporters play a major role in maintaining cellular ion homeostasis under salinity stress. Therefore the expression profile of *a priori* candidates for ion homeostasis with significant effects were further examined (**Figure 5**, **Supplementary Figure S5**). We provide a potential model of transporter activity and divergence in **Figure 6**.

Compartmentalization of Na^+^ ions into vacuoles by tonoplast localized Na^+^/H^+^ exchanger (NHX) like antiporters is considered as a key mechanism to avoid the toxic effects of Na^+^ in the cytosol both in root and leaf tissues (Apse et al., 1999; Apse and Blumwald, 2007). Among the three annotated *NHX2* homologs (PhHAL.2G474100, PhHAL.3G288900 and PhHAL.8G243100) in the *P. hallii* var HAL2 reference genome, two were differentially expressed in this experiment. PhHAL.3G288900 was upregulated in leaf tissue for the inland genotype under salinity stress, whereas the coastal genotype had higher expression in root tissue constitutively. The other *NHX2* detected in this study (PhHAL.2G474100) was upregulated in leaf tissue for both genotypes during salinity treatment. This compartmentalization of Na^+^ by NHX is driven by the proton-motive force generated by the vacuolar H^+^-ATPase (V-H^+^-ATPase) and the plastic response of V-H^+^-ATPase has been reported during salinity stress (Lv et al., 2017). A vacuolar ATPase (PhHAL.5G103300) was upregulated during salinity treatment in root tissue for both genotypes. Overall, we observed constitutive expression of vacuolar antiporter in root tissue for the coastal genotype but the expression was higher in leaf tissue for the inland genotype. In leaf tissue, plasma membrane-bound Na^+^/H^+^ exchanger (SOS1) (Qiu et al., 2002; Ji et al., 2013) and high affinity potassium transporter (HKT) (Davenport et al., 2005; Davenport et al., 2007) play an active role in loading Na^+^ into phloem recirculation and shuttle Na^+^ from shoot tissue to maintain ion homeostasis. *SOS1* (PhHAL.3G490000) and *HKT1* (PhHAL.4G025000) were upregulated in leaf tissue for both the genotypes under salinity stress and are perhaps involved in Na^+^ recirculation from leaf tissue to phloem. Overall, *NHX2* was upregulated in a genotype and tissue specific manner whereas *SOS1* and *HKT1* demonstrated leaf specific upregulation irrespective of genotype.

KT/HAK/KUP family transporters help to maintain K^+^ homeostasis by driving K^+^ uptake from the soil (Wang et al., 2022). In root tissue one *KT* transporter (PhHAL.2G242200) was upregulated during salinity in the coastal genotype while a *HAK* (PhHAL2G.001500) transporter was upregulated during salinity stress irrespective of genotype. However, the upregulation of several *KT* (PhHAL.2G242200) and *KUP* (PhHAl.2G471600, PhHAL.5G026500) transporters in leaf tissue in the inland genotype suggest their possible involvement in maintaining cytoplasmic K^+^ levels when external Na^+^ inhibits K^+^ uptake and cellular Na^+^ replaces K^+^ (Maathuis, 2005). Inward rectifying potassium channel (KAT) expressed in epidermal cell can mediate K^+^ uptake (Obata et al., 2007). *KAT1* (PhHAL.1G101800) was upregulated in the coastal genotype during salinity treatment and could be involved in K^+^ uptake in leaf tissue.

Outward rectifying K^+^ channels (KCO) activated by membrane depolarization due to high Na^+^ influx can release K^+^ from the vacuolar pool to cytosol or mediate K^+^ exclusion across the plasma membrane (Voelker et al., 2006; Shabala and Cuin, 2008; Kumari et al., 2021). Two KCO genes (PhHAL.9G082300 and PhHAL.9G082500) were both upregulated under salinity stress for the inland genotype in leaf. The presence of these two KCO family genes in the QTL intervals for qK-9@17.2, qNa-9@17.2, and qNa/K-9@17.2 suggests a plausible link between their activity and the imbalance of sodium ion homeostasis. Overall, we detected upregulation of genes involved in K^+^ acquisition from soil in root tissue for the coastal genotype, genotype specific upregulation of genes responsible for K^+^ influx in leaf tissue, and upregulation of several potassium channels in the inland genotype responsible for K^+^ efflux in leaf tissue during salinity treatment. Taken together the expression profiles of various Na^+^ and K^+^ transporters in the coastal genotype demonstrated overexpression of genes associated with Na^+^ compartmentalization and K^+^ acquisition in root tissue. Conversely, the inland genotype exhibited overexpression of genes which mediates Na^+^ compartmentalization and both influx and efflux of K^+^ in leaf tissue in the face of salinity (**Figure 6**).

## 4 Discussion

In this study we sought to understand the physiological and growth response variation of coastal and inland ecotypes of *P. hallii* under salinity stress and identify the genetic basis of traits associated with salinity adaptation. We also conducted genome wide gene expression studies to test whether differential transcriptional responses contribute to these adaptive physiological responses. Growth stability and superior ion homeostasis of coastal genotypes was detected during salinity treatment and several genetic loci associated with these responses were identified. Genome wide transcriptional profiles revealed various ion transporters were differentially upregulated in the coastal genotype and could be potential candidates contributing to the maintenance of superior ion homeostasis.

Under a model of local adaptation, we hypothesized that coastal genotypes would outperform inland genotypes in the presence of salinity treatment. We detected aboveground and belowground growth and biomass stability in response to salinity for plants collected from coastal habitats compared to inland habitats which support our hypothesis. This is consistent with previous report that the salt tolerant genotypes of Proso millet, another *Panicum* species, had higher aboveground and belowground biomass accumulation compared to its sensitive counterparts (Yuan et al., 2022). Studies have reported that salt adapted genotypes tend to maintain a lower Na^+^/K^+^ (Platten et al., 2013; Tang et al., 2013; Assaha et al., 2017) and some tolerant genotypes demonstrated no significant change in leaf Na^+^ content while still maintaining lower Na^+^/K^+^ compare to sensitive genotypes (Sun et al., 2015). Our physiological study revealed lower leaf Na^+^ and superior maintenance of leaf ion homeostasis (low Na^+^/K^+^) in coastal genotypes compared to inland genotypes. This implies that the coastal population might have evolved efficient Na^+^ exclusion and K^+^ retention as a mechanism to maintain ion homeostasis.

The detectable divergence in leaf ion homeostasis between coastal and inland genotypes and moderate heritability in the RIL mapping population (narrow-sense heritability, h^2^ ~24 -36%) implies the presence of substantial genetic variation in this population in response to salinity. We detected several QTL for Na^+^, K^+^, and Na^+^/K^+^. The majority of these QTL effects were driven by the coastal alleles contributing superior ion homeostasis (low Na^+^, high K^+^, or low Na^+^/K^+^). Moreover, we noticed enrichment of *a priori* salinity tolerant candidate genes such as the HKT gene family (Davenport et al., 2005; Ren et al., 2005; Davenport et al., 2007) in some ionic QTL intervals. However, these detected ionic QTL are not directly associated with the plastic ionic response but correspond to treatment specific response. Overall, this study provides evidence of existing adaptive genetic variation in *P. hallii* for leaf ion homeostasis.

Overexpression of various cation transporters have been reported to confer salinity tolerance. For example, overexpression of *NHX* mediates Na^+^ compartmentalization while overexpression of KT/KUP/HAK gene family members regulates K^+^ acquisition and certain K^+^ channels regulating ion homeostasis and leads to salinity tolerance in diverse plant species (Roy et al., 2014; Kumari et al., 2021). Several cation transporters were enriched in differentially expressed genes for the GxT category and exhibited tissue specific expression differences. For instance, a putative *NHX* (PhHAL.3G112000) was upregulated in leaf tissue for the inland genotype under salinity but it was constitutively overexpressed in root tissue for the coastal genotype irrespective of treatment conditions. The constitutive expression pattern of *NHX* in root tissue suggests that the coastal genotype might be primed for grown in a saline habitat. In addition, *KT* (PhHAL.2G242200) was upregulated in the coastal genotype relative to the inland genotype during salinity treatment in root tissue. Another K^+^ channel (KAT1) (PhHAL.1G101800) was upregulated in the coastal genotype compared to the inland genotype during salinity treatment in leaf tissue. Conversely, two outward rectifying K^+^ channels (*KCO*) (PhHAL.9G082300 and PhHAL.9G082500) were upregulated during salinity treatment in the inland genotype in leaf tissue. It is likely that the coastal population may have evolved to strictly regulate the expression of various ion transporters to retain potassium and regulate cytosolic ion homeostasis. Moreover, these two *KCO* family genes were detected as candidate genes in one QTL cluster (qK-9@17.2, qNa-9@17.2, and qNa/K-9@17.2) for which the coastal allele contributed to superior leaf ion homeostasis. Among these two KCO family genes, PhHAL.9G082500 had elevated non-synonymous to synonymous substitution rate (dN/dS = 1.52) and all four nonsynonymous codon substitution were derived in the coastal genotype (orthologs from *Panicum virgatum* were used to infer the ancestral state) implying that this gene might be under positive selection in the coastal lineage and could contribute to efficient K^+^ retention in these putatively adapted genotypes.

This study aimed to investigate the response of coastal and inland genotypes to salinity and therefore was restricted to experiments in controlled growth conditions. However, the effect of this response towards the relative fitness of individuals in their native habitats can be altered by the spatiotemporal magnitude of soil salinity and through complex interaction with other environmental factors. Therefore, this study cannot directly infer the role of soil salinity as a driving force for local adaptation. However, it can provide some basic insight into physiological functions under stress and serve as the basis for developing hypotheses concerning local adaptation. For instance, in an earlier study on *P. hallii* Lovell et al. (2018) reported genetic clusters for drought responsive gene expression in a field experiment and demonstrated more plastic drought recovery responses of the inland genotype. In our genetic mapping we detected one QTL cluster for which inland alleles contributed to superior leaf ion homeostasis. It is possible that the inland populations might have evolved to maintain ion homeostasis using different sets of genetic loci compared to coastal salt**-**exposed populations. However, it is also reasonable to infer that some of the molecular mechanisms maintaining ion homeostasis could be the result of crosstalk between shared pathways for salinity and water stress (Zhu, 2002). Moreover, we noticed that the confidence interval of the responsive QTL associated with belowground biomass (qBGB-3@15.8) overlapped (~86% of the confidence interval) with one of the reported trans-eQTL hotspots with genotype specific drought response by Lovel et al. (2018). This interval was enriched with genes related to molecular function such as oxidoreductive and antioxidant activity. It is possible that candidate genes within this interval have a role in general stress responses associated with scavenging reactive oxygen species (ROS) (Golldack et al., 2014; You and Chan, 2015). Detection of genomic regions related to both salinity and water stress supports the idea that some convergent molecular mechanisms for adaptation to abiotic stresses might be present in divergent locally adapted populations of *P. hallii*.

In summary, our study demonstrates that coastal *P. hallii* genotypes have superior performance in response to salinity treatment compared to inland genotypes and maintained stable growth and better ion homeostasis. We identified genetic loci associated with growth maintenance and ion homeostasis, and several differentially expressed candidate genes associated with these traits are included various ion transporters. These findings improve our understanding of molecular mechanisms underlying local adaptation to saline habitats. Broadly, natural genetic variants identified for ion homeostasis could provide potential resources for functional validation of these candidates by genetic manipulation.

## Data availability statement

The datasets presented in this study can be found in online repositories or in **Supplementary Material**. The name of the repository and accession numbers can be found below: NCBI Sequence Read Archive (SRA) database under the Bioproject PRJNA853054.

## Author contributions

TH and TJ conceived the project and designed the research; TH with the help of GB, JY, and JB performed the research. TH analyzed the data. TH and TJ wrote the article, with input from GB. All authors contributed to the article and approved the submitted version.

## Funding

The research was supported by NSF Plant Genome Research Program (Award: IOS-1444533) and Integrative Biology Research Grant for graduate students, UT Austin. This research was also supported by the US Department of Energy, Office of Science, Office of Biological and Environmental Research, Genomic Science Program grant DE-SC0021126 to TJ.

## Acknowledgments

We thank the lab members of the Juenger Lab for helping with phenotypic data collection and Shane Merrell for his support while conducting experiment in UT greenhouse facilities. We also thank Robert Heckman and Zeba I. Seraj for their constructive comments on this manuscript.

## Conflict of interest

The authors declare that the research was conducted in the absence of any commercial or financial relationships that could be construed as a potential conflict of interest.

## Publisher’s note

All claims expressed in this article are solely those of the authors and do not necessarily represent those of their affiliated organizations, or those of the publisher, the editors and the reviewers. Any product that may be evaluated in this article, or claim that may be made by its manufacturer, is not guaranteed or endorsed by the publisher.
