# Supplementary material for: Natural variation in growth and leaf ion homeostasis in response to salinity stress in Panicum hallii
Source: Front Plant Sci. 2022 Oct 7;13:1019169. doi: 10.3389/fpls.2022.1019169 (PMC9586453; doi:10.3389/fpls.2022.1019169)
Supplement: Supplementary file 1 [file DataSheet_1.docx]

**Supplementary method S1**

**List of Supplementary:**

Supplementary Figure S1: Representative image of inland coastal *P. hallii* population

Supplementary Figure S2: Conditional LOD profile plots of detectable QTLs in constitutive, responsive, or ionic categories

Supplementary Figure S3: Effect plot for the epistatic interaction between qK-T-1@10 and qK-T-9@17.2

Supplementary Figure S4: Global PCA plot of all TAGSeq libraries including leaf and root tissues

Supplementary Figure S5: Gene expression profile of selected ion transporters in GxT category

Supplementary Table S1: Geographical location of *P. hallii* plant materials used in Experiment 1

Supplementary Table S2: Summary statistics of Experiment 1

Supplementary Table S3: Summary statistics of Experiment 2

Supplementary Table S4: Summary statistics of Parents in Experiment 3

Supplementary Table S5: Phenotypes of RIL mapping population

Supplementary Table S6: Narrow-sense heritability of RIL mapping population

Supplementary Table S7: Detailed statistics for QTL models

Supplementary Table S8: Sequencing statistics of TAGSeq libraries

Supplementary, Table S9: List of DEGs in leaf tissue

Supplementary, Table S10: GO enrichment analysis for different categories of DEGs in leaf tissue

Supplementary, Table S11: List of DEGs in root tissue

Supplementary, Table S12: GO enrichment analysis for different categories of DEGs in root tissue

Supplementary Method S1: Supplementary with detailed methods

**1. Methods**

**1.1 Measurement of soil salinity**

The soil salinity at three coastal and two inland sites were measured on equally mixed proportion of soil samples (0-15 cm depth) collected from a given site. Soil salinity test was performed on saturated paste extract by the Soil, Water, and Forage Testing Laboratory at Texas A&M University ([http://soiltesting.tamu.edu](http://soiltesting.tamu.edu/))

**1.2 Composition of Nutrient Solution**

Dyna-Gro Grow 7-9-5 (https://dyna-gro.com/product/liquid-grow/) was diluted as recommended for hydroponic non-recirculating systems and was used as a nutrient solution. This full-strength solution composed of 9% Phosphorus, 7% Nitrogen, 5% Potassium, 2% Calcium, 0.5% Magnesium, 0.1% Chlorine, 0.1% Iron, 0.1% Sodium, 0.05% Copper, 0.05% Sulphur, 0.02% Boron, 0.0015% Cobalt, 0.05% Manganese, 0.05% Zinc, 0.0009% Molybdenum and 0.0001% Nickel. The percentages are in weight by volume unit.

**1.3 Methods to measure Above ground Biomass (AGB) and Below ground Biomass (BGB)**

To measure AGB, all aboveground tissue samples were harvested, bagged, oven dried at 65°C for 72 h and later the dry weight was recorded. To measure below ground biomass first pots with root tissues were placed in cold storage to prevent degradation prior to processing. Later, all roots were gently harvested from each pot, cleaned to remove soil, bagged, and oven dried at 65°C for 72 h.

**1.4 Methods to measure leaf Relative Water Content (RWC)**

In order to estimate leaf relative water content, we harvested a fully expanded 2nd leaf from each plant and immediately measured the fresh weight (FW) of the leaf. Subsequently, the petiole of this leaf was dipped into a 15 ml tube containing 2 ml of water, the tube was sealed carefully with a cotton ball without damaging the leaf structure and left overnight to hydrate. The next day we measured the hydrated weight (HW) of the leaf and then dried each leaf in a 65 °C oven for 48 hours. Later the dried weight (DW) of the leaf was measured and RWC was estimated using the following equation:

$$RWC (\%)=100 \times\frac{HW-FW}{HW-DW}$$

**1.5 Methods to measure Osmotic Potential**

To measure the osmolarity we followed the leaf extraction sap method (Callister et al. 2006). A fully expanded leaf was flash frozen in liquid nitrogen and stored at -20 °C. Later this frozen leaf was thawed, and approximately 10 cm segments was used to extract leaf sap. Each sample segment was further chopped in 2.5 cm small segments, placed into a 0.5 mL tube with a bottom slit and was allowed to be absorb moisture for 2-4 hours in a warm cooler with steam for rehydration. Later this tube was placed in a 1.5 mL tube and was centrifuged at 13,000 RPM for 5 minutes to collect leaf sap. Osmolarity (c) of each sample was determined with 10 μL of leaf sap using a vapor pressure osmometer (Vapro 5520, Wescor, USA). Later, osmotic potential was derived by Van't Hoff equation.

**1.6 Methods to measure Leaf Sodium and Potassium**

We measured leaf Na and K ion content following the protocol described at Munns et al (2010). In brief, we harvested the third leaf of plants, washed with Milli-Q water (18 ohm) to remove possible external ion contamination and dried at 65°C for 48 h. Later this dried leaf was ground to fine powder and carefully weighted (allowed weight range: 8 mg -12 mg) in glass vial using a five-digit balance and digested in 5 ml 0.5 M Nitric acid in an 80°C oven for 2 h. Subsequently, vials were centrifuged at 4000 rpm and clear supernatant was collected and was further diluted to measure the ion concentration by Inductively Coupled Plasma Optical Emission Spectrometry (ICP-OES) using Varian 710-ES instrument (Agilent Technologies, Santa Clara, CA). ICP-OES was performed on samples in batches (batch sample size=50) and each batch had: i) a set of ionic standards, ii) sample blanks and ii) three diluted independent digestions of Standard Reference Material® NIST SRM 1570a Spinach Leaf as a reference. Ion concentration was measure using calibration curve method and all regression coefficients for calibration curves for different batches had R^2^ > 0.99. Possible measurement drift by the machine between batches was normalized by the ratio of expected concentration of a given ion to the mean estimated concentration of that ion in the spinach leaf for a given batch. We rerun a batch if the difference between expected concentration of this reference and the mean concentration estimated exceeded >5%. Finally, leaf ion content (umol per gm dry weight) was measure by the following equation:

$$ion content=\frac{Measured Ion concentration \left( ug/mL \right)x Sample Volume(mL) X Dilution factor}{Dry weight of leaf \left( in gm \right) X Molecular weight of the ion}$$

**1.7 TAGSeq library construction and count data generation for global gene expression study**

We extracted total RNA from leaf and root using the Trizol (invitrogen) method and used 1ug of RNA sample to construct 3′ TAGSeq libraries as described by Weng et al (2019). These libraries were sequenced using 1x150 bp single-end reads on HiSeq2500 for an average yield of 5 million reads per library. Raw reads were quality filtered (average quality>20 and minimum length=70 bp) and trimmed for adapter sequences and homopolymers (polyA or polyT for base count of >=15mer) using Cutadapt (Martin 2011). Reads which passed this quality control were mapped to *P. hallii* var. HAL2 reference genome (v2.0 ) using the BWA-mem algorithm (Li,Durbin 2009). Alignments were filtered for read quality ( MAPQ >=10) with SAMtools (Li et al. 2009) and duplicated alignments were marked by Picard (<http://broadinstitute.github.io/picard>). We used Feature counts to generate a count matrix using the v2.1 gene models as reference (--readExtension3 0 –readExtention5 0 -d 50 -s 1). Raw reads were deposited in NCBI Sequence Read Archive (SRA) as Bioproject PRJNA853054. Detail information for sequencing, coverage, quality, and mapping statistics are provided in Supplementary, Table S7).

**1.8 Differential Gene Expression analysis**

Due to high divergence between leaf and root tissue expression patterns we carried out subsequent expression analyses separately for each tissue type. Genes with mean count >4 across all samples for a given tissue were included in subsequent DEG analysis. First, the libraries were normalized for their library size by scaling counts with their corresponding estimated size factors using the sizeFactors function and normalized count data was transformed with a variance stabilizing transformation (VST) using the fitted dispersion-mean relationship.  The normalized read counts for genes were used for Discriminant analysis of principle component (DAPC) to visualize the global variation in gene expression between different levels of genotype x treatment combinations (four groups) using adegenet package (Jombart et al. 2010). Plasticity of treatment was defined as the Euclidean distance (of first two linear discriminatory axes) from each biological replicate at a given genotype in salinity treatment group to the mean of the control group for that given genotype. Later, we implemented Likelihood Ratio Tests (LTR) from the DESeq2 package in R to test for differentially expressed genes (DEG) at genotype (G), treatment (T) and ecotype x treatment (GxT) levels (Love et al. 2014). For each expressed gene, this test compares the full model with corresponding reduced models by dropping the model term of interest. We used a false-discovery rate (FDR) method to account for multiple testing and consider an adjusted p-value < 0.05 as significant. Subsequently, we used the variancePartition package (Hoffman,Schadt 2016) on the VST transformed count data for a given tissue to partition the variance explained by each fixed effect (G, T and GxT) across all the expressed genes.

**1.9 Gene Ontology Enrichment test:**

Gene Ontology (GO) enrichment was tested for different sets of targets using the topGo package (Adrian Alexa,Rahnenfuhrer 2021). We used a multiple test correction based on the FDR method and GO terms with adjusted p-value < 0.1 were considered significant. For the GO enrichment analysis of different categories of DEG, we considered only the expressed transcriptome for a given tissue as the background for enrichment analysis while we considered all the annotated genes for *P. hallii* var. HAL2 reference genome (v2.1 annotation) as background for candidate genes of a given QTL interval.

**1.10 QTL mapping and Heritability estimation:**

We used a linkage map of 901 evenly distributed markers for QTL mapping analysis. A detailed description of the linkage map construction for this RIL mapping population has been described at Dryad (<https://doi.org/10.5061/dryad.73n5tb2w8>). All the traits except Na, K and Na/K were measured for both control and salinity treatment level. We implemented a QTL analysis framework based on the mean across treatments (control + salinity/2) (“constitutive” category) and difference (control - salinity) (“responsive” category) of AGB, BGB, RB and LRWC. The traits related to leaf ion homeostasis (K, Na, Na/K) were measure only for salinity treated plants for one cohort. Prior to QTL mapping, normality of trait distributions were tested by “shapiro.test” function in R using Shapiro-Wilk normality test method and were power transformed by Box-Cox power transformation method (Box,Cox 1964) if required. We used the “scanone” function in the R/qtl(Broman et al. 2003) package to identify QTL for constitutive and responsive category traits with the following models:

QTL_Constitutive_ : Y_mean_ (mean of control and stress for each RIL) = µ + QTL + cohort (if applicable) + QTL x cohort (if applicable)  *+ error*

QTL_Responsive_ : Y_Diff_ (Difference from Stress to Control) = µ + QTL + cohort (if applicable) + QTL x cohort (if applicable)  *+ error*

To test for the significance of cohort and QTL x cohort effect, we fit the following models for each constitutive and responsive category trait and obtained the null distribution of each model by performing 1000 permutation using a constant seed at the beginning of the permutation process. This way the null distribution of each model was comparable for a given trait.

Full model, F: Y = µ + QTL + cohort + QTL x cohort *+ error*

Reduced models:

Additive-cohort model, R_1_: Y = µ + QTL + cohort *+ error*

Non-cohort model, R_2_: Y = µ + QTL *+ error*

Next, we compared the null distribution of full model (F) with reduced model R_1_ by calculating the difference of LOD scores and obtained the LOD thresholds for the difference of these two models (Type I error=0.05). If the differences of LOD score of full QTL model to the reduced model) passed the threshold then it was considered to have significant effect of QTL x cohort interaction. Similarly, we compared model R_1_ with R_2_. Traits that demonstrate significant additive effect of cohort, we retained cohort as an additive covariate for subsequent analysis.

The following model was implemented for Na, K and Na/K to detect QTL:

QTL_Ionic_ : Y_Treat_ (Trait measure in treatment condition) = µ + QTL  *+ error*

Subsequently, to detect potential epistasis interaction the “scantwo” function was implemented. We performed permutations (n=1000) for each trait using “scantwo” function which was stratified by cohort to obtain the null distribution for main and epistasis interaction effects. The stepwise QTL function was used to scan using a forward-backward search by adding/dropping QTL effects and their epistasis effect and were allowed to fit n+2 number of QTL (n=number of QTL found by “scanone” function). The threshold value for Type I error was set as 0.1 for all the traits and QTL intervals were calculated with a threshold of 1.5 LOD drop from the peak. Estimated QTL effects were obtained by “qtlStats” function from qtlTools package (Lovell 2018) with the final QTL model for a given trait. The assumption of normality for the residuals of QTL models were examined by Q-Q plot.

Narrow-sense heritability for a given trait of a given category (constitutive, responsive or ionic) was defined as a proportion of phenotypic variance explained by the additive kinship matrix of the mapping population and was estimated by using “est_herit” function from qtl2 package by fitting a mixed model including the cohort as covariate when it was appropriate.

**1.11 Enrichment test for a priori genes**

To address whether we observe a priori genes responsible for salinity tolerance more frequently within QTL intervals we performed a test to compare the frequency of any of this a priori gene family in a given QTL interval with any random genomic background region which had the same length of QTL interval. We first obtained a list of genes annotated as HKT gene family (Davenport et al. 2005; Davenport et al. 2007; Ren et al. 2005) , genes in SOS pathway (Møller et al. 2009; Qiu et al. 2002; Ji et al. 2013), or KT/KUP/HAK family of potassium transporter genes (Shen et al. 2015; Chen et al. 2015; Obata et al. 2007) in *P. hallii* var. HAL2 reference genome (v2.1 annotation). For a given QTL interval we counted the frequency (f_0_) of one given gene family. We randomly sampled a genetic interval of the same length of the given QTL interval which did not overlap with this QTL interval and considered this as background interval. Next, we counted the frequency (f) of the given gene family in the background interval and repeated this process for 1000 iterations. The p-value was defined by as the ratio of the number of iterations which had frequency >= f_0_ by the total number of iterations (1000).

p-value = ⅀(f >= f_0_)/N

**1.12 Estimate dN/dS across coastal and inland lineages**

In order to estimate dN/dS of outward rectifying K^+^ channels (KCO) gene (PhHAL.9G082500), we first obtained the protein and coding sequence of the ortholog gene pair from inland and coastal reference ecotype. These protein sequences were first aligned with clustal-omega (Thompson et al. 1994) tool and subsequent codon-based alignment of nucleotide sequences was performed by PAL2NAL (Suyama et al. 2006). dN/dS was estimated using CODEML function of PAML V4.9 (runmode -2, F3X4 codon frequency) (Yang 2007). The ancestral state of protein sequence variants between coastal and inland ecotype was inferred using *Panicum virgatum* orthologs (Pavir.9KG462010 and Pavir.9KG461696) as reference.

**REFERENCES:**

Adrian Alexa, Rahnenfuhrer, J.: topGO: Enrichment Analysis for Gene Ontology. R package version 2.46.0. (2021)

Box, G.E.P., Cox, D.R.: An Analysis of Transformations. Journal of the Royal Statistical Society. Series B (Methodological) **26**(2), 211-252 (1964)

Broman, K.W., Wu, H., Sen, Ś., Churchill, G.A.: R/qtl: QTL mapping in experimental crosses. Bioinformatics **19**(7), 889-890 (2003). doi:10.1093/bioinformatics/btg112

Callister, A.N., Arndt, S.K., Adams, M.A.: Comparison of four methods for measuring osmotic potential of tree leaves. Physiologia Plantarum **127**(3), 383-392 (2006). doi:<https://doi.org/10.1111/j.1399-3054.2006.00652.x>

Chen, G., Hu, Q., Luo, L., Yang, T., Zhang, S., Hu, Y., Yu, L., Xu, G.: Rice potassium transporter OsHAK1 is essential for maintaining potassium-mediated growth and functions in salt tolerance over low and high potassium concentration ranges. Plant, Cell & Environment **38**(12), 2747-2765 (2015). doi:<https://doi.org/10.1111/pce.12585>

Davenport, R., James, R.A., Zakrisson-Plogander, A., Tester, M., Munns, R.: Control of Sodium Transport in Durum Wheat  Plant Physiology **137**(3), 807-818 (2005). doi:10.1104/pp.104.057307

Davenport, R.J., Alicia, M.-M., Jha, D., Essah, P.A., Rus, A., Tester, M.: The Na+ transporter AtHKT1;1 controls retrieval of Na+ from the xylem in Arabidopsis. Plant, Cell & Environment **30**(4), 497-507 (2007). doi:<https://doi.org/10.1111/j.1365-3040.2007.01637.x>

Hoffman, G.E., Schadt, E.E.: variancePartition: interpreting drivers of variation in complex gene expression studies. BMC Bioinformatics **17**(1), 483 (2016). doi:10.1186/s12859-016-1323-z

Jombart, T., Devillard, S., Balloux, F.: Discriminant analysis of principal components: a new method for the analysis of genetically structured populations. BMC Genetics **11**(1), 94 (2010). doi:10.1186/1471-2156-11-94

Li, H., Durbin, R.: Fast and accurate short read alignment with Burrows–Wheeler transform. Bioinformatics **25**(14), 1754-1760 (2009). doi:10.1093/bioinformatics/btp324

Li, H., Handsaker, B., Wysoker, A., Fennell, T., Ruan, J., Homer, N., Marth, G., Abecasis, G., Durbin, R., Subgroup, G.P.D.P.: The Sequence Alignment/Map format and SAMtools. Bioinformatics **25**(16), 2078-2079 (2009). doi:10.1093/bioinformatics/btp352

Love, M.I., Huber, W., Anders, S.: Moderated estimation of fold change and dispersion for RNA-seq data with DESeq2. Genome Biology **15**(12), 550 (2014). doi:10.1186/s13059-014-0550-8

Lovell, J.T.: qtlTools. <https://github.com/jtlovell/qtlTools> (2018).

Martin, M.: Cutadapt removes adapter sequences from high-throughput sequencing reads. 2011 **17**(1), 3 (2011). doi:10.14806/ej.17.1.200

Munns, R., Wallace, P.A., Teakle, N.L., Colmer, T.D.: Measuring Soluble Ion Concentrations (Na+, K+, Cl−) in Salt-Treated Plants. In: Sunkar, R. (ed.) Plant Stress Tolerance: Methods and Protocols. pp. 371-382. Humana Press, Totowa, NJ (2010)

Obata, T., Kitamoto, H.K., Nakamura, A., Fukuda, A., Tanaka, Y.: Rice Shaker Potassium Channel OsKAT1 Confers Tolerance to Salinity Stress on Yeast and Rice Cells. Plant Physiology **144**(4), 1978-1985 (2007). doi:10.1104/pp.107.101154

Ren, Z.-H., Gao, J.-P., Li, L.-G., Cai, X.-L., Huang, W., Chao, D.-Y., Zhu, M.-Z., Wang, Z.-Y., Luan, S., Lin, H.-X.: A rice quantitative trait locus for salt tolerance encodes a sodium transporter. Nature Genetics **37**(10), 1141-1146 (2005). doi:10.1038/ng1643

Shen, Y., Shen, L., Shen, Z., Jing, W., Ge, H., Zhao, J., Zhang, W.: The potassium transporter OsHAK21 functions in the maintenance of ion homeostasis and tolerance to salt stress in rice. Plant, Cell & Environment **38**(12), 2766-2779 (2015). doi:<https://doi.org/10.1111/pce.12586>

Suyama, M., Torrents, D., Bork, P.: PAL2NAL: robust conversion of protein sequence alignments into the corresponding codon alignments. Nucleic Acids Research **34**(suppl_2), W609-W612 (2006). doi:10.1093/nar/gkl315

Thompson, J.D., Higgins, D.G., Gibson, T.J.: CLUSTAL W: improving the sensitivity of progressive multiple sequence alignment through sequence weighting, position-specific gap penalties and weight matrix choice. Nucleic Acids Research **22**(22), 4673-4680 (1994). doi:10.1093/nar/22.22.4673

Weng, X., Lovell, J.T., Schwartz, S.L., Cheng, C., Haque, T., Zhang, L., Razzaque, S., Juenger, T.E.: Complex interactions between day length and diurnal patterns of gene expression drive photoperiodic responses in a perennial C4 grass. Plant, Cell & Environment **42**(7), 2165-2182 (2019). doi:<https://doi.org/10.1111/pce.13546>

Yang, Z.: PAML 4: Phylogenetic Analysis by Maximum Likelihood. Molecular Biology and Evolution **24**(8), 1586-1591 (2007). doi:10.1093/molbev/msm088
