# Supplementary material for: Natural variation in growth and leaf ion homeostasis in response to salinity stress in Panicum hallii
Source: Front Plant Sci. 2022 Oct 7;13:1019169. doi: 10.3389/fpls.2022.1019169 (PMC9586453; doi:10.3389/fpls.2022.1019169)
Supplement: Supplementary file 2 [file DataSheet_2.docx]

Supplementary Figure S1: Representative figure of coastal (left: FIL2) and inland (right: HAL2) genotypes of *Panicum hallii* grown in control growth condition.


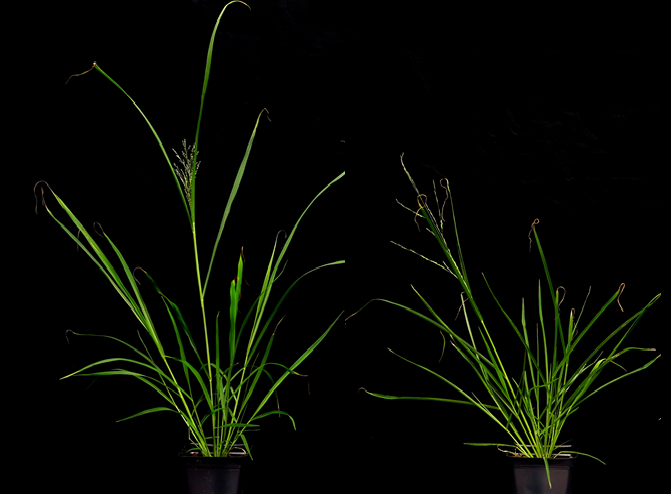


Supplementary Figure S2: Conditional LOD profile plots of detectable QTLs in constitutive, responsive, or ionic categories. Y-axis represents the conditional LOD score for QTL and x-axis repents genetic distance of markers across at a given chromosome.

A) Conditional LOD profile plots of detectable constitutive QTLs for Aboveground Biomass (AGB)


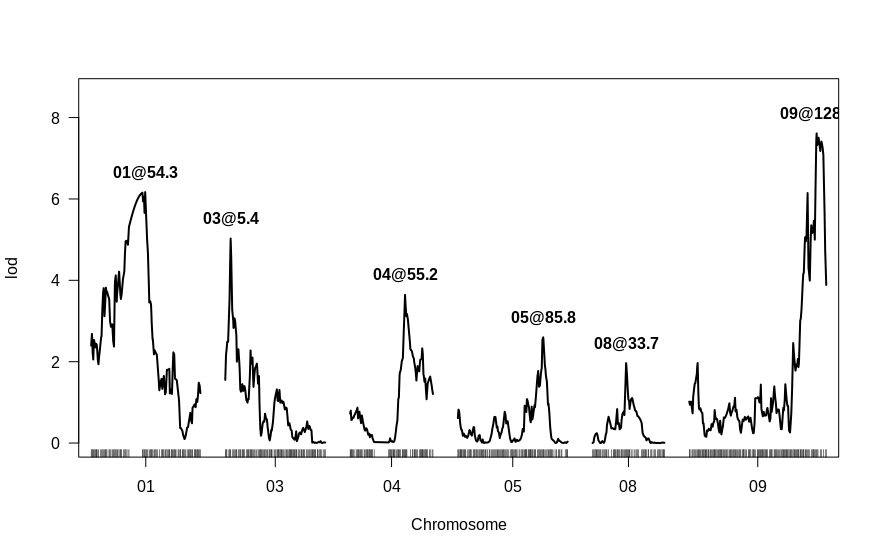


B) Conditional LOD profile plots of detectable constitutive QTLs for Belowground Biomass (BGB)


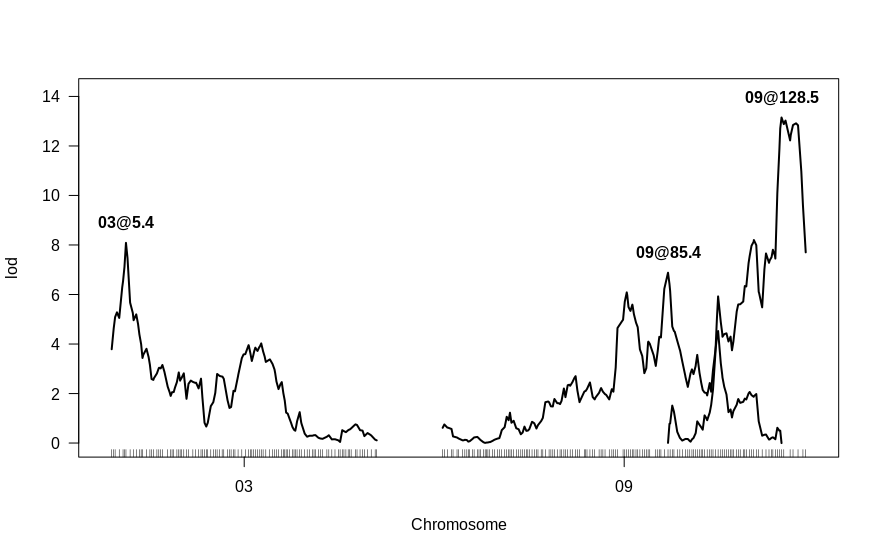


C) Conditional LOD profile plots of detectable constitutive QTLs for Ration of Biomass (RB)


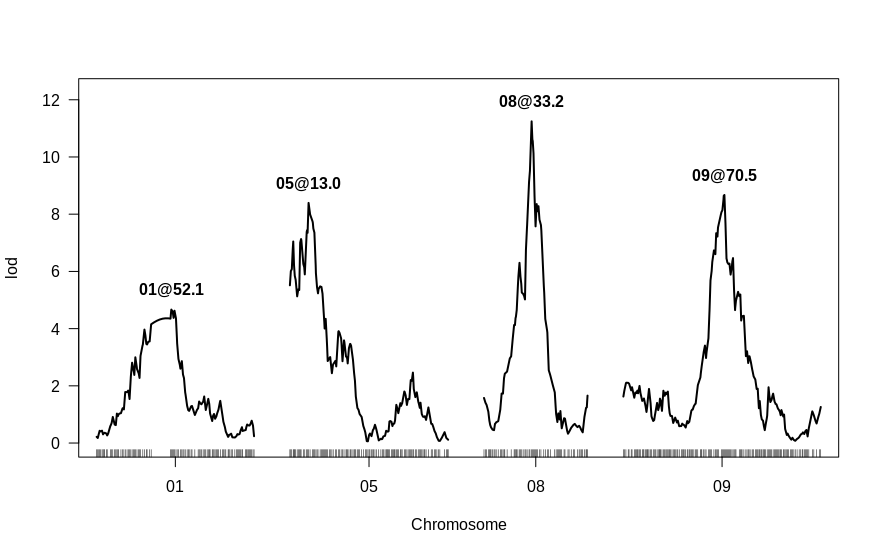


D) Conditional LOD profile plots of detectable responsive QTLs for Belowground Biomass (BGB)


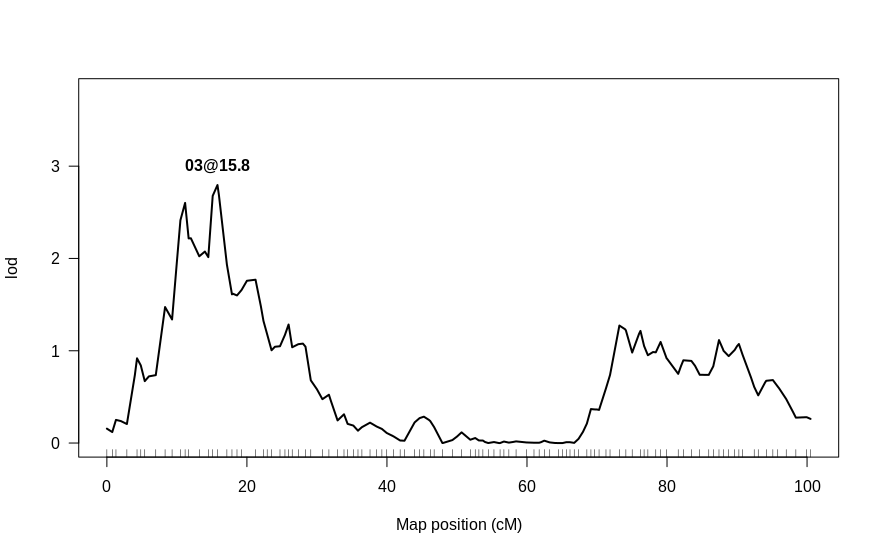


E) Conditional LOD profile plots of detectable constitutive QTLs for Leaf Potassium (K^+^)


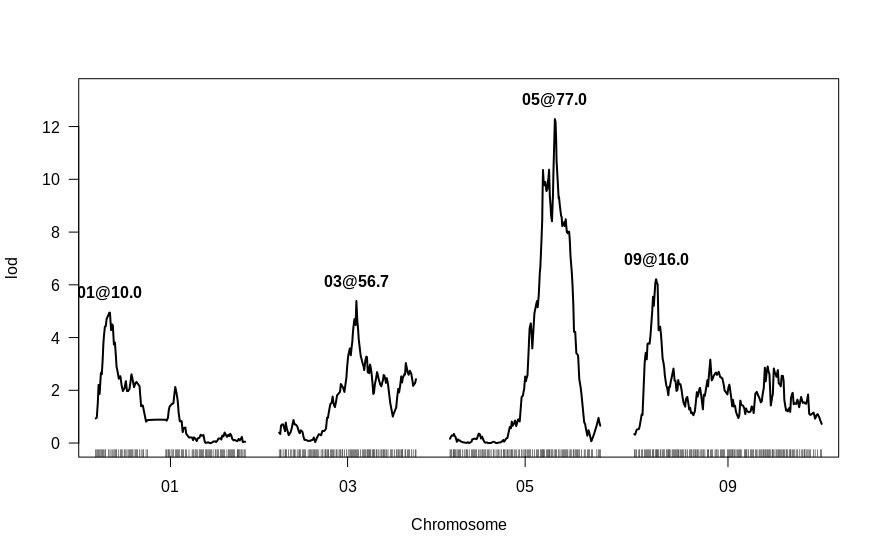


F) Conditional LOD profile plots of detectable constitutive QTLs for Leaf Sodium (Na^+^)


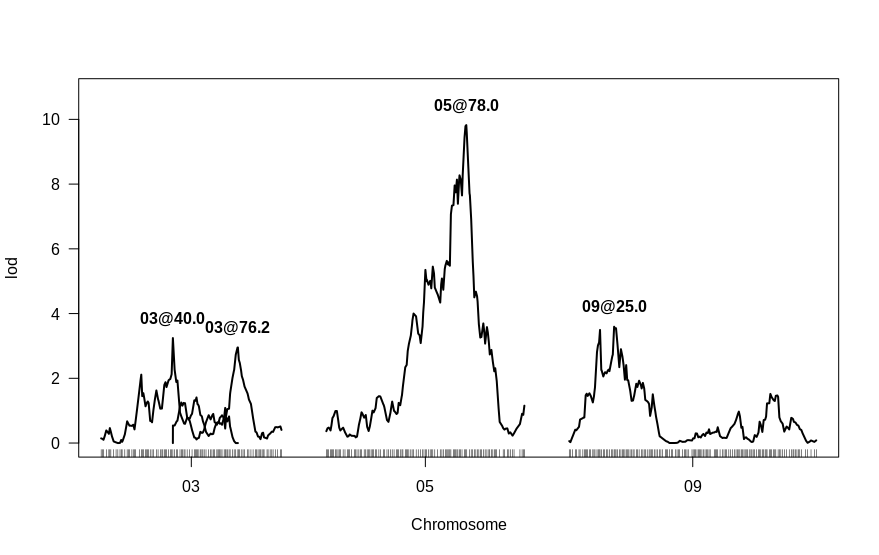


G) Conditional LOD profile plots of detectable constitutive QTLs for the ratio of Sodium to Potassium (Na^+^/K^+^)


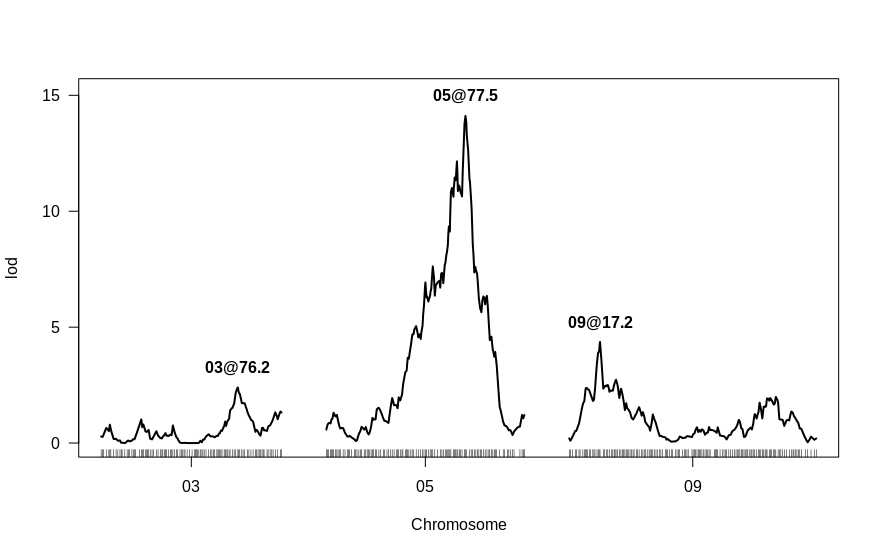


Supplementary Figure S3: Effect plot for the epistatic interaction between qK-T-1@10 and qK-T-9@17.2


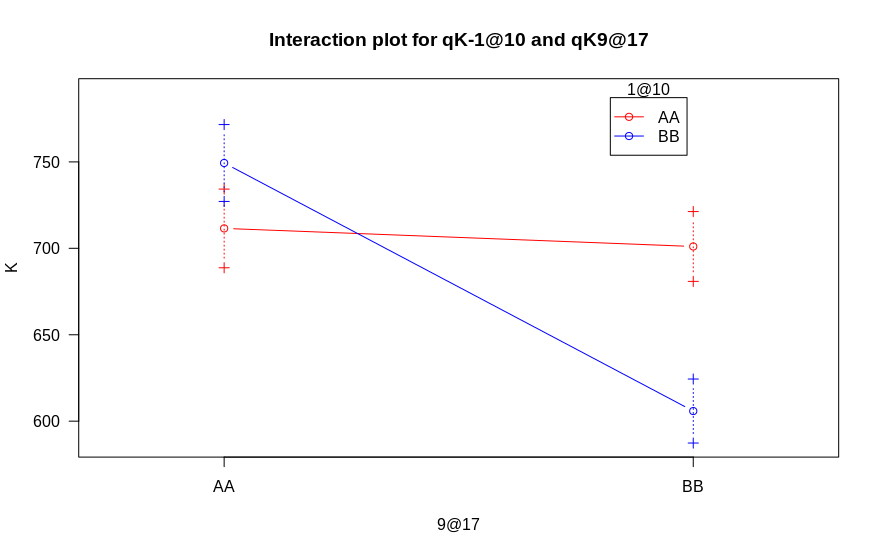


Interaction plots of allelic effect of the epistatic interaction between qK-T-1@10 and qK-T-9@17.2. Blue line represents plants with the inland allele (BB) at qK-T-1@10 whereas the red line indicates plants with the coastal allele (AA). Alleles at qK-T-9@17.2 are plotted on x-axis for which AA and BB indicate the coastal and inland allele respectively. Open points represent group-wise allelic mean and dotted lines represent corresponding Standard Error (SE).

Supplementary Figure S4: Global PCA plot of all TAGSeq libraries including leaf and root tissues


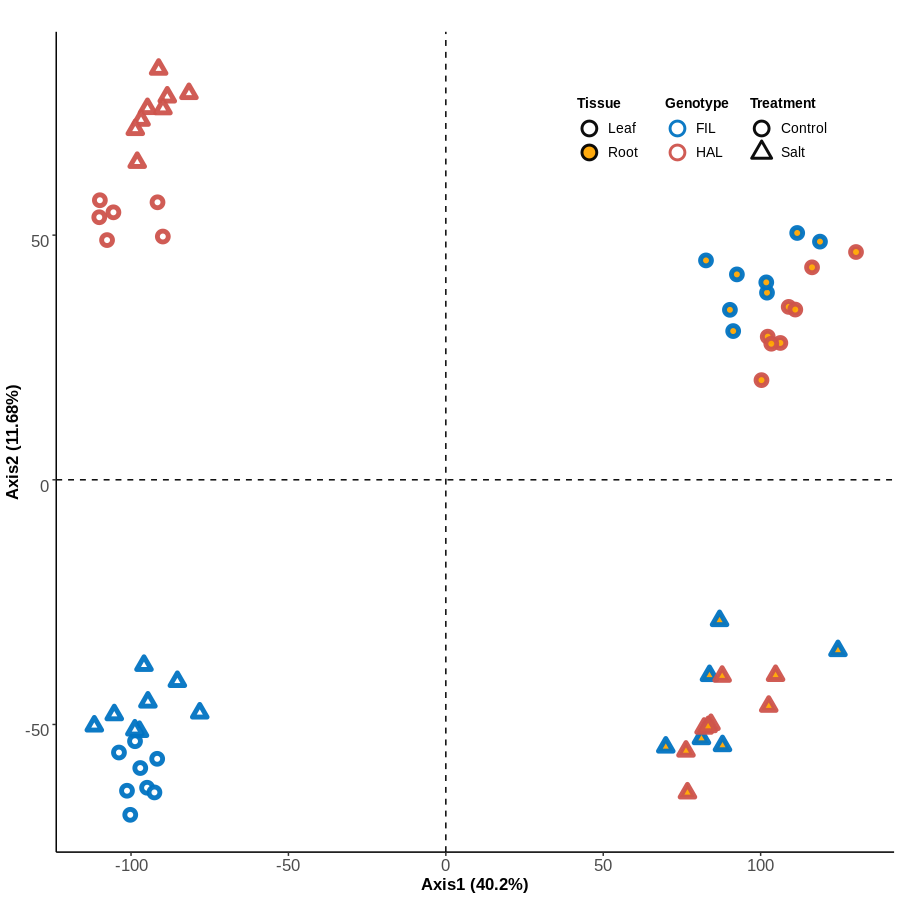


Global PCA (Principle component analysis) plot summering all TAGSeq libraries including the leaf and root tissues. Axis 1 and 2 represent the first and second Principle components for the analysis. Each data point represents one TAGSeq library. Open points represent the leaf tissue while filled points (with orange color) represent the root tissue. Points are color coded by genotype (blue=coastal genotype and red=inland genotype). Shape of the points depicts different stress levels (circle=control and triangle=salinity)
