# Supplementary material for: Natural variation in growth and leaf ion homeostasis in response to salinity stress in Panicum hallii
Source: Front Plant Sci. 2022 Oct 7;13:1019169. doi: 10.3389/fpls.2022.1019169 (PMC9586453; doi:10.3389/fpls.2022.1019169)
Supplement: Supplementary file 3 [file DataSheet_3.pdf]

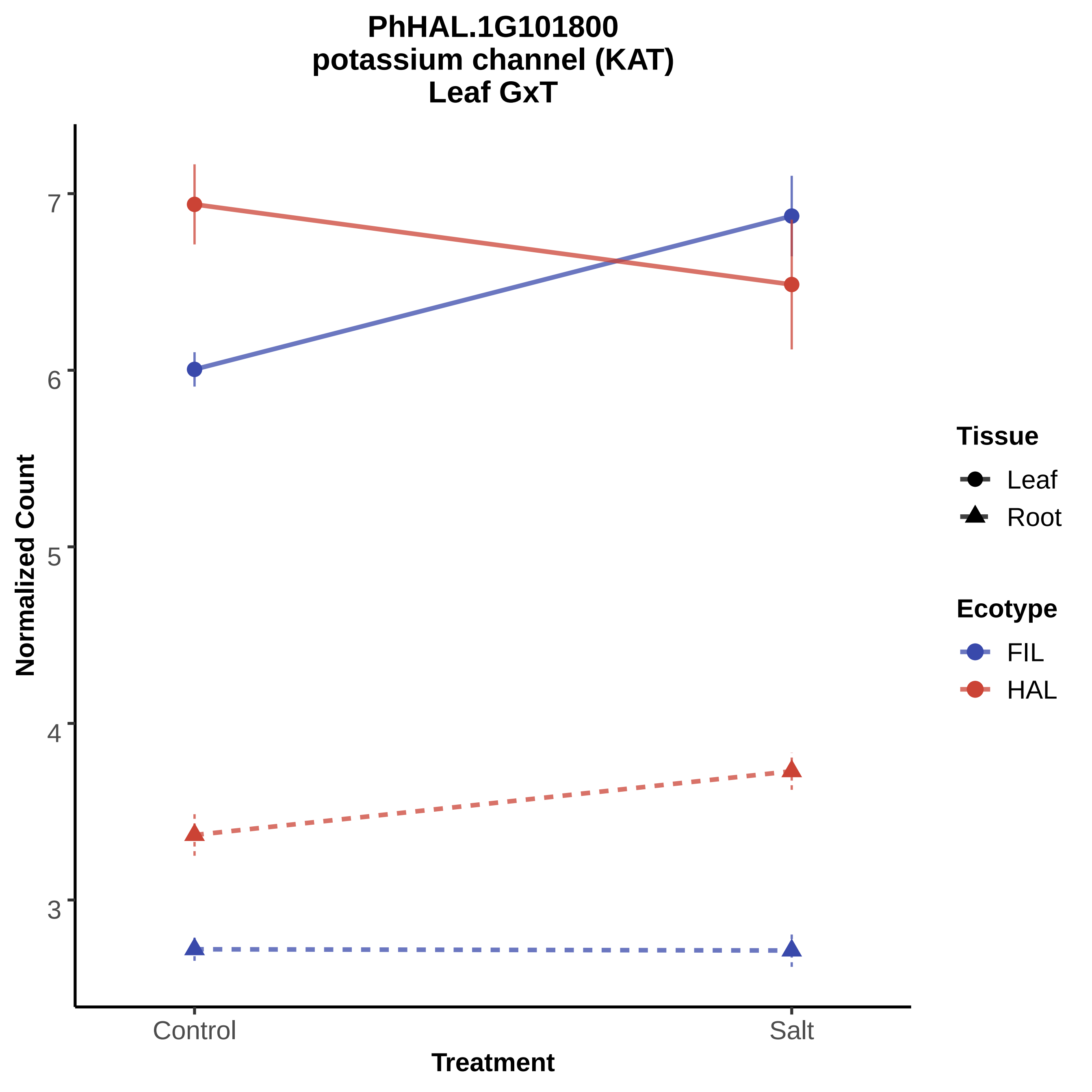
PhHAL.1G101800
potassium channel (KAT)
Leaf GxT
Normalized Count
7
6
5
4
3
Control
Salt
Treatment
Tissue
Leaf
Root
Ecotype
FIL
HAL

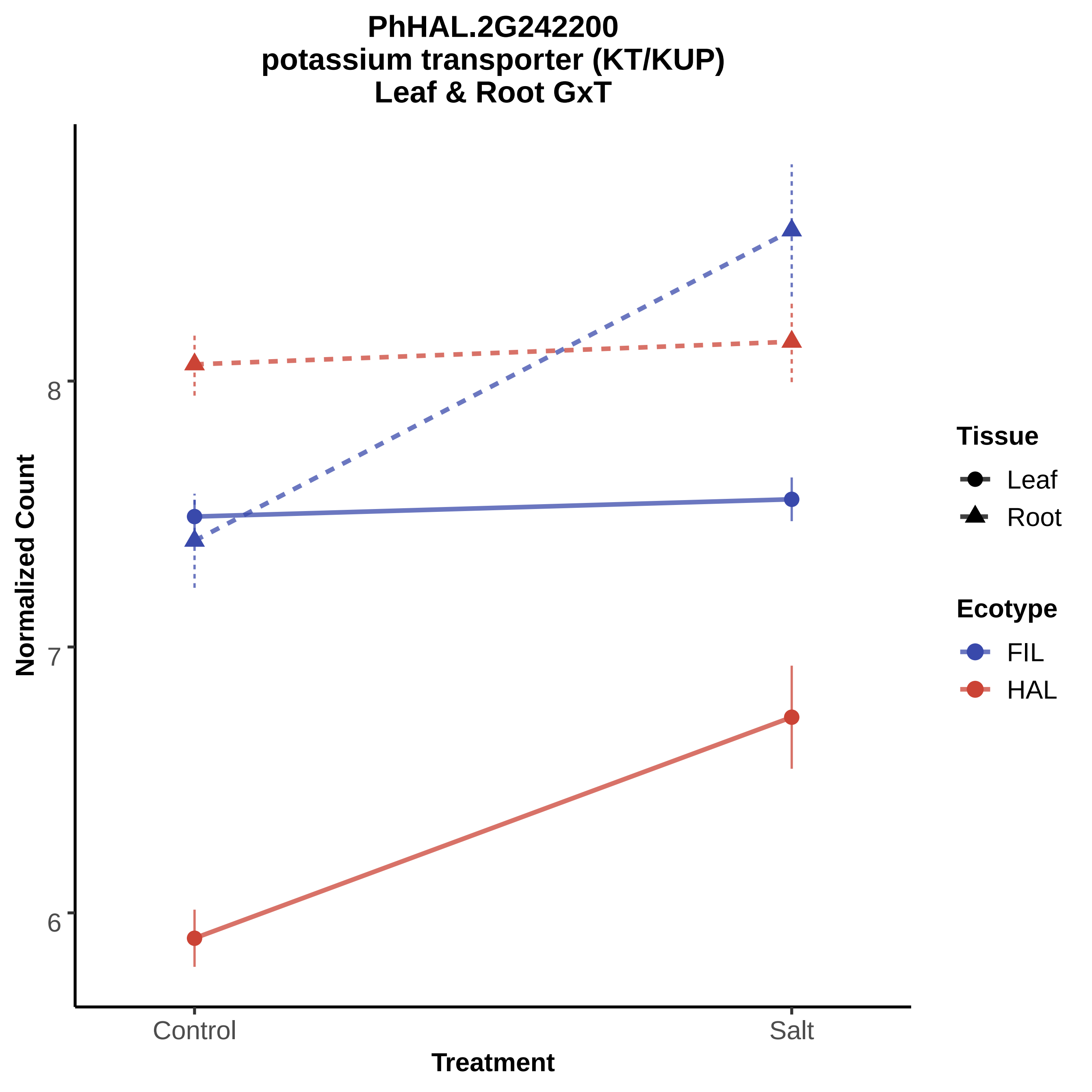
PhHAL.2G242200
potassium transporter (KT/KUP)
Leaf & Root GxT
Normalized Count
8
7
6
Control
Salt
Treatment
Tissue
Leaf
Root
Ecotype
FIL
HAL

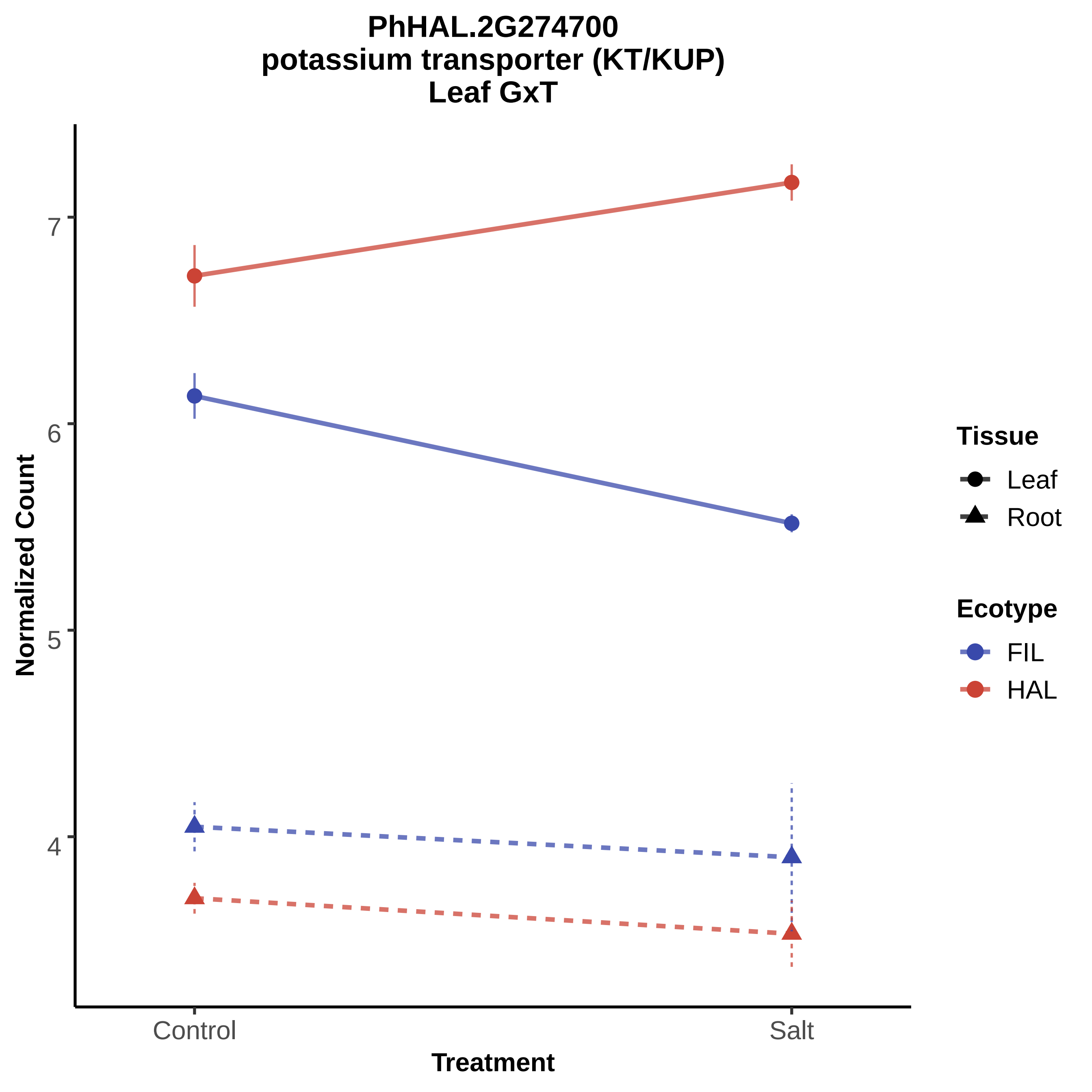
PhHAL.2G274700
potassium transporter (KT/KUP)
Leaf GxT
Normalized Count
7
6
5
4
Control
Salt
Treatment
Tissue
Leaf
Root
Ecotype
FIL
HAL

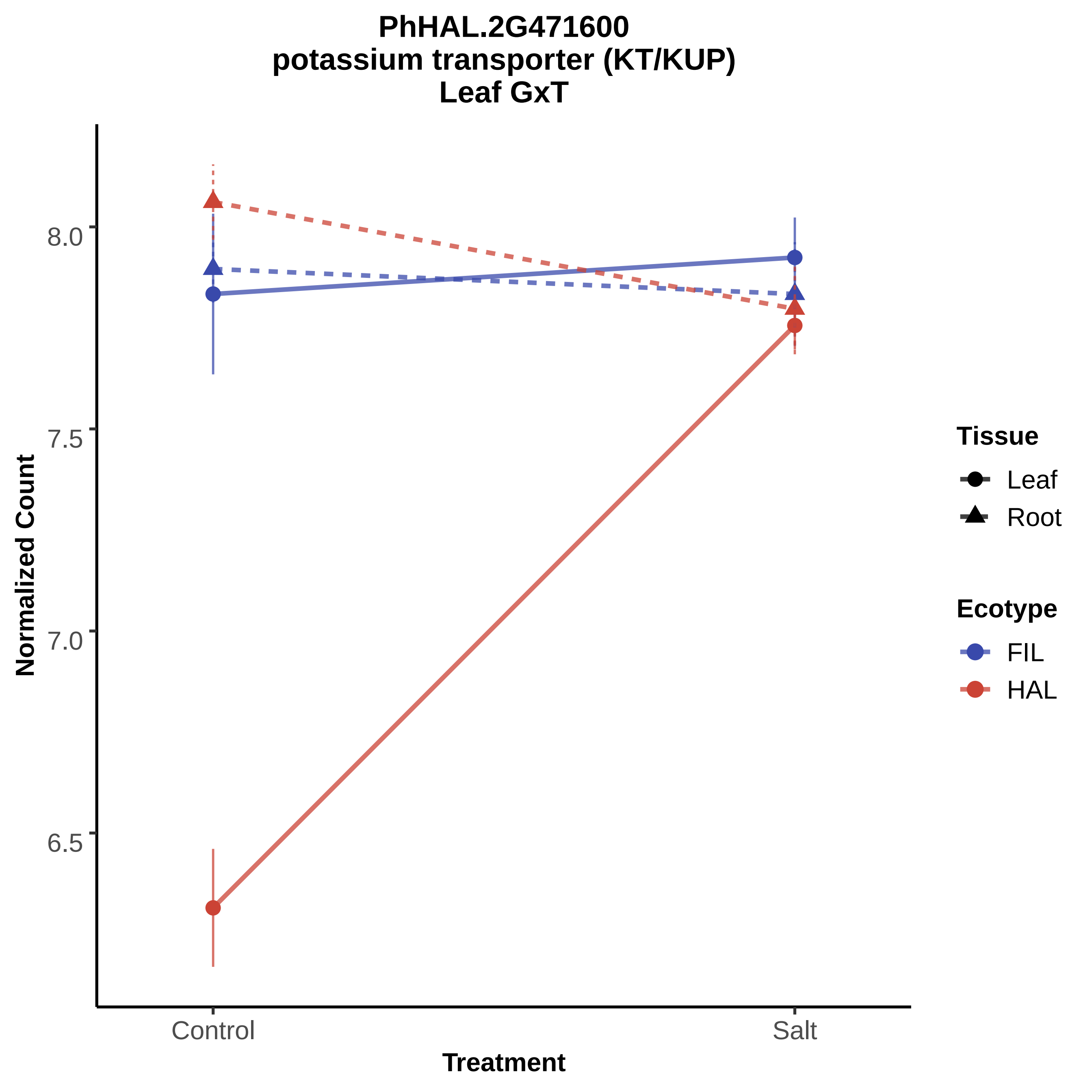
PhHAL.2G471600
potassium transporter (KT/KUP)
Leaf GxT
Normalized Count
8.0
7.5
7.0
6.5
Control
Salt
Treatment
Tissue
Leaf
Root
Ecotype
FIL
HAL

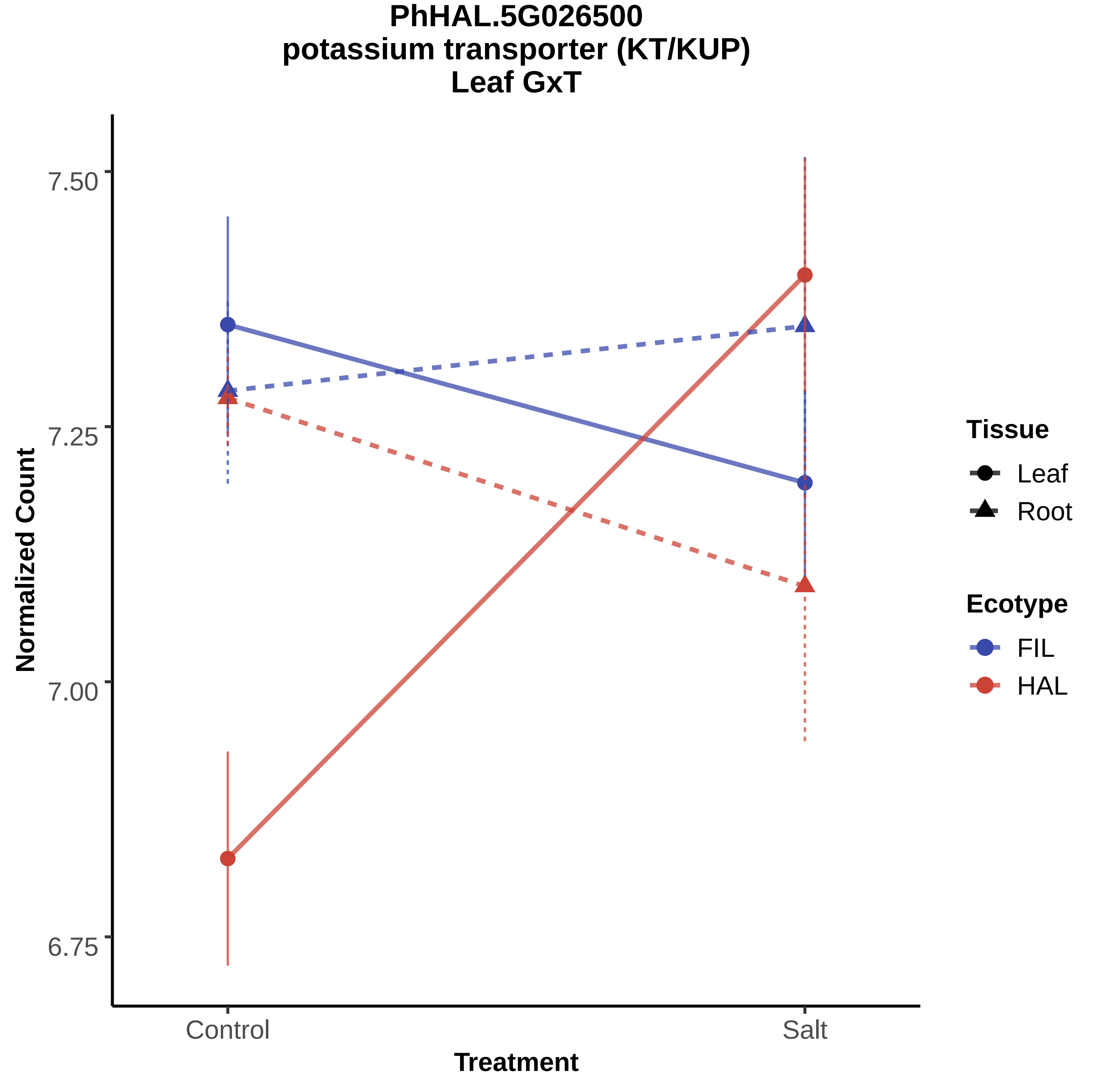
PhHAL.5G026500
potassium transporter (KT/KUP)
Leaf GxT
Normalized Count
7.50
7.25
7.00
6.75
Control
Salt
Treatment
Tissue
Leaf
Root
Ecotype
FIL
HAL

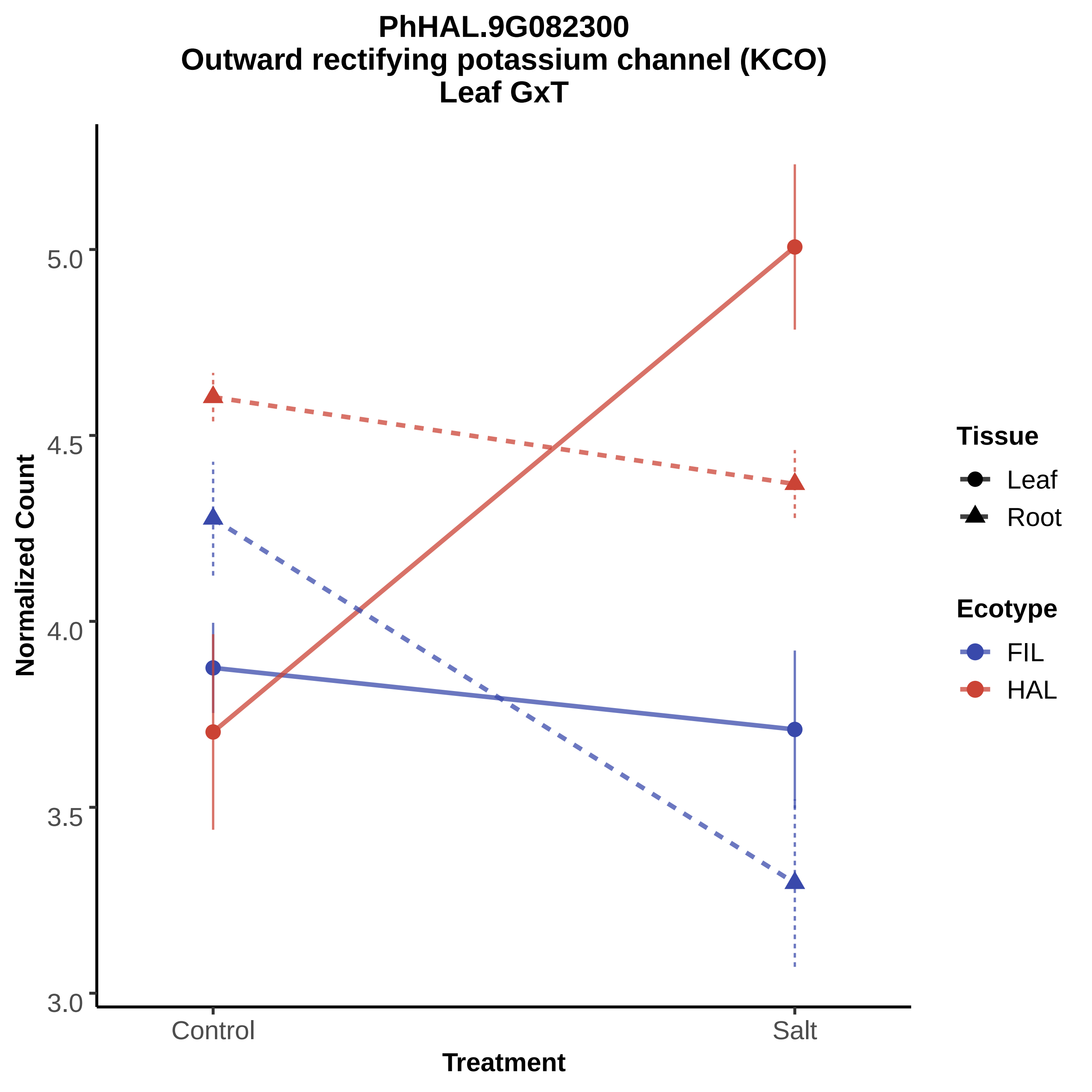
PhHAL.9G082300
Outward rectifying potassium channel (KCO)
Leaf GxT
Normalized Count
5.0
4.5
4.0
3.5
3.0
Control
Salt
Treatment
Tissue
Leaf
Root
Ecotype
FIL
HAL

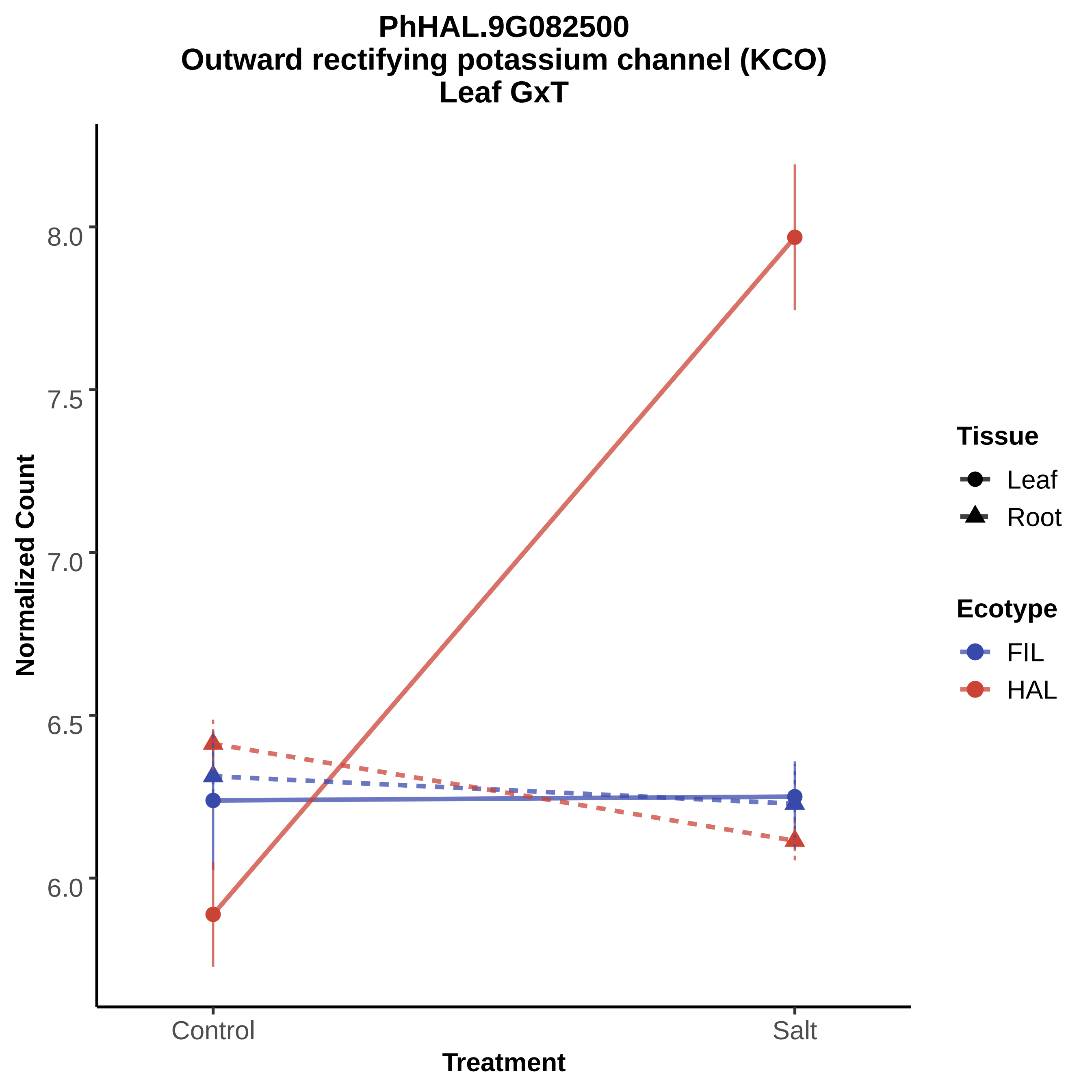
PhHAL.9G082500
Outward rectifying potassium channel (KCO)
Leaf GxT
Normalized Count
8.0
7.5
7.0
6.5
6.0
Control
Salt
Treatment
Tissue
Leaf
Root
Ecotype
FIL
HAL

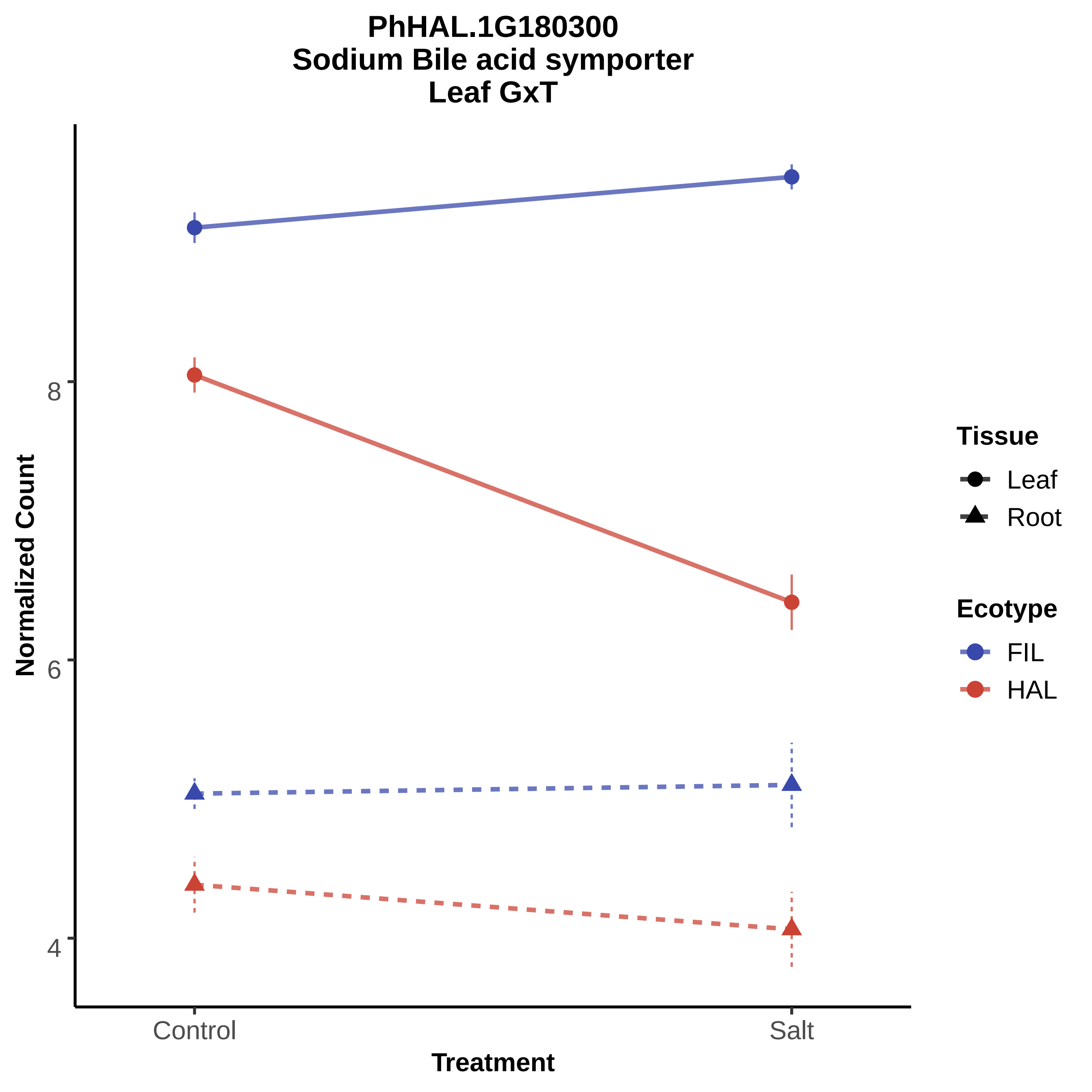
PhHAL.1G180300
Sodium Bile acid symporter
Leaf GxT
Normalized Count
8
6
4
Control
Salt
Treatment
Tissue
Leaf
Root
Ecotype
FIL
HAL

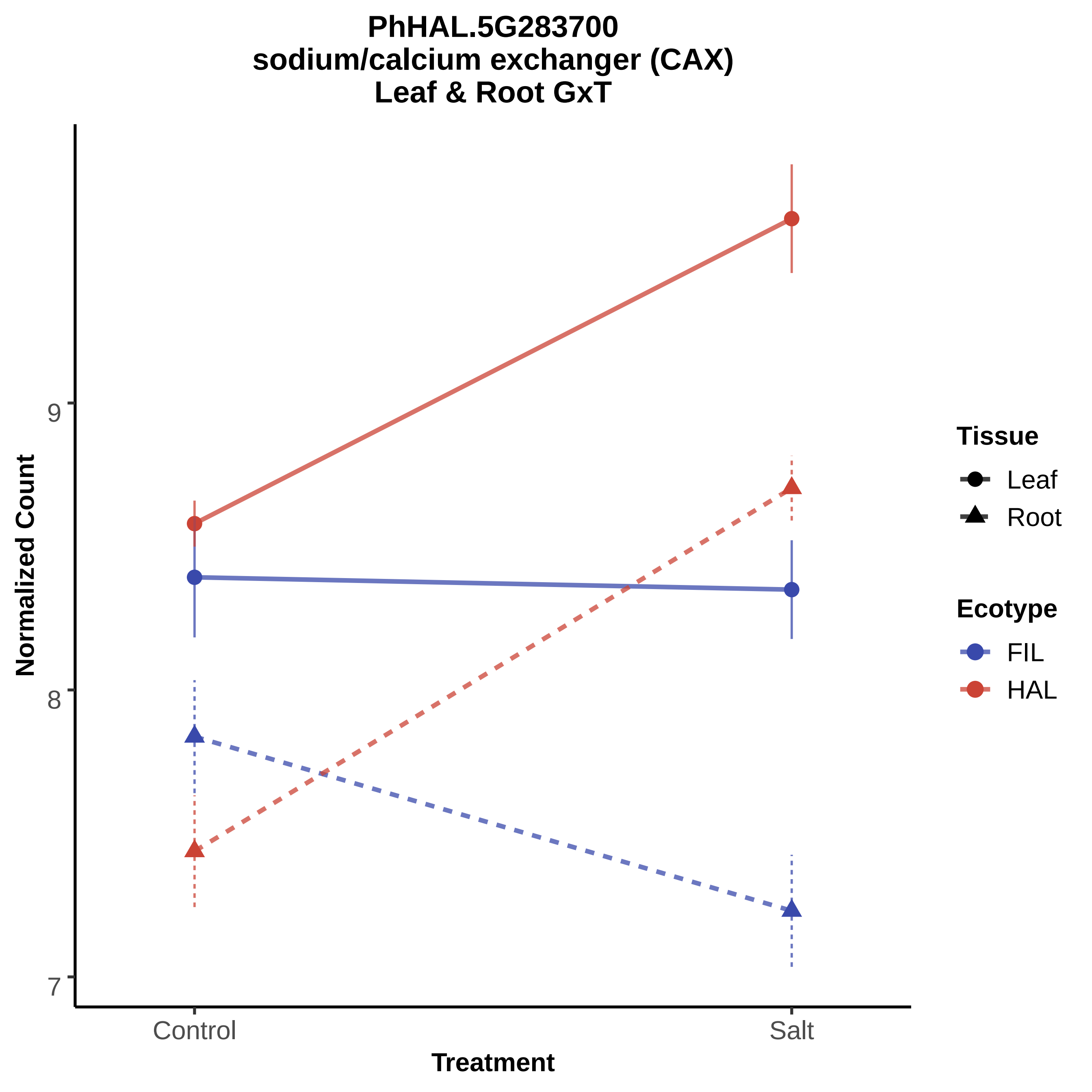
PhHAL.5G283700
sodium/calcium exchanger (CAX)
Leaf & Root GxT
Normalized Count
9
8
7
Control
Salt
Treatment
Tissue
Leaf
Root
Ecotype
FIL
HAL

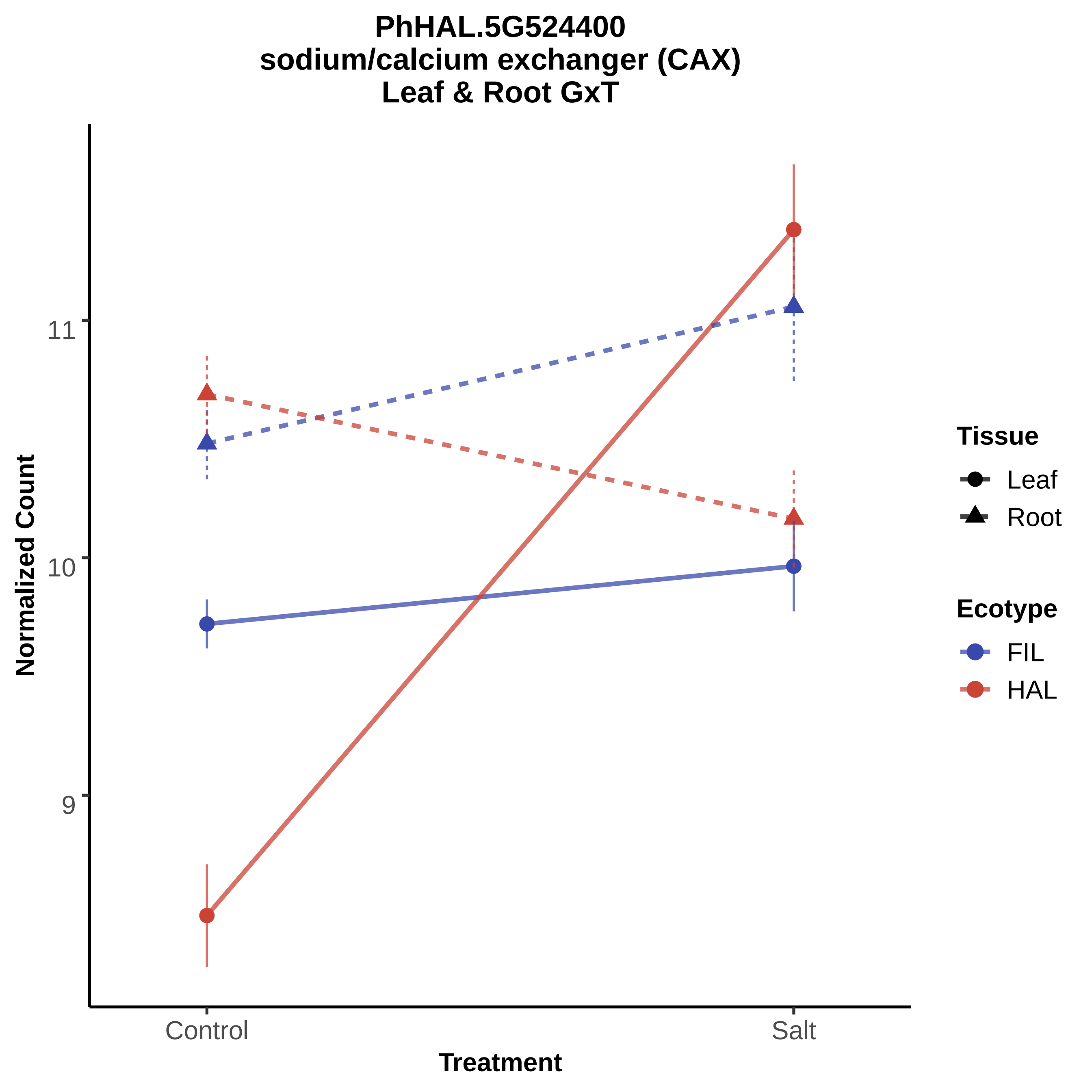

PhHAL.5G524400
sodium/calcium exchanger (CAX)
Leaf & Root GxT
Normalized Count
11
10
9
Control
Salt
Treatment
Tissue
Leaf
Root
Ecotype
FIL
HAL

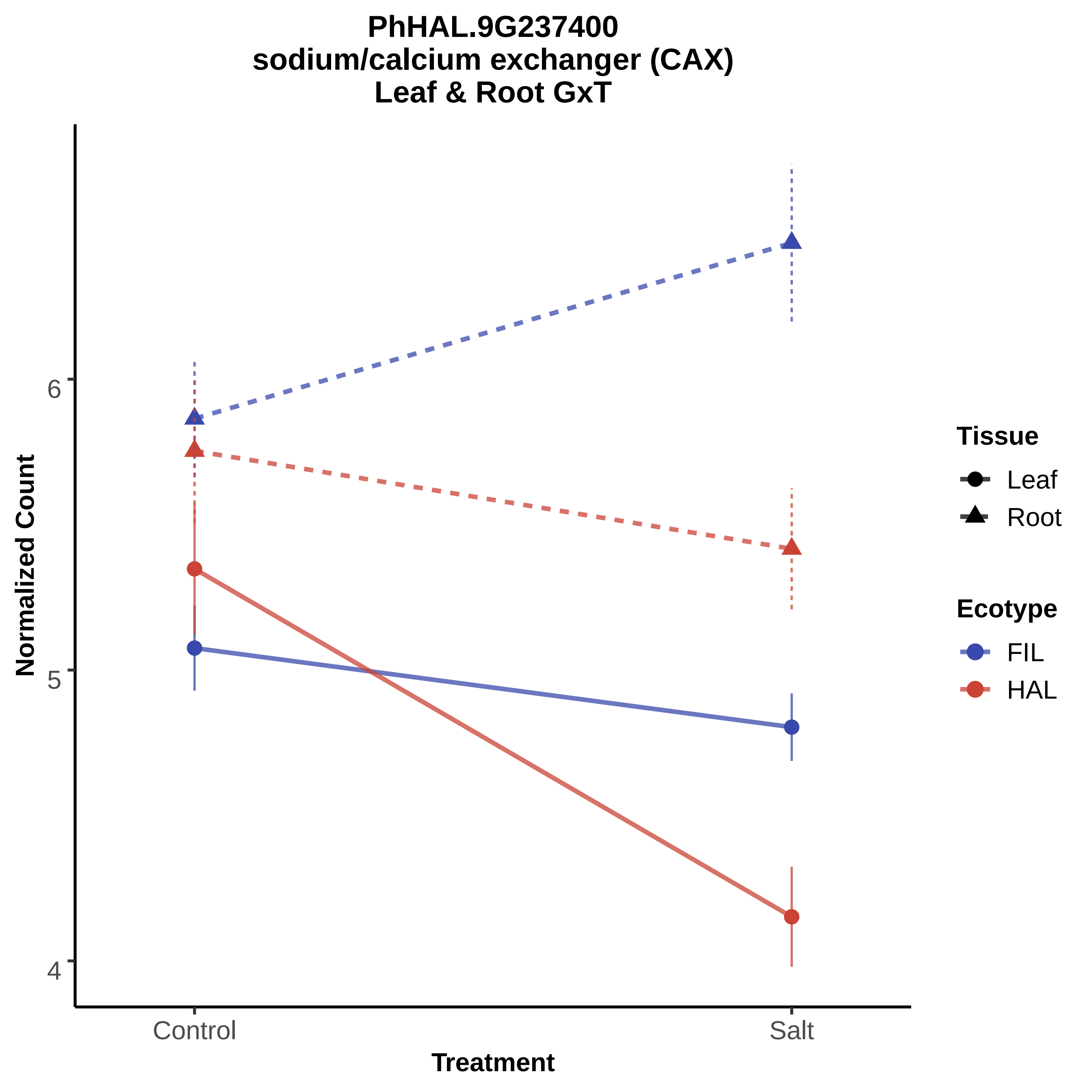
PhHAL.9G237400
sodium/calcium exchanger (CAX)
Leaf & Root GxT
Normalized Count
6
5
4
Control
Salt
Treatment
Tissue
Leaf
Root
Ecotype
FIL
HAL

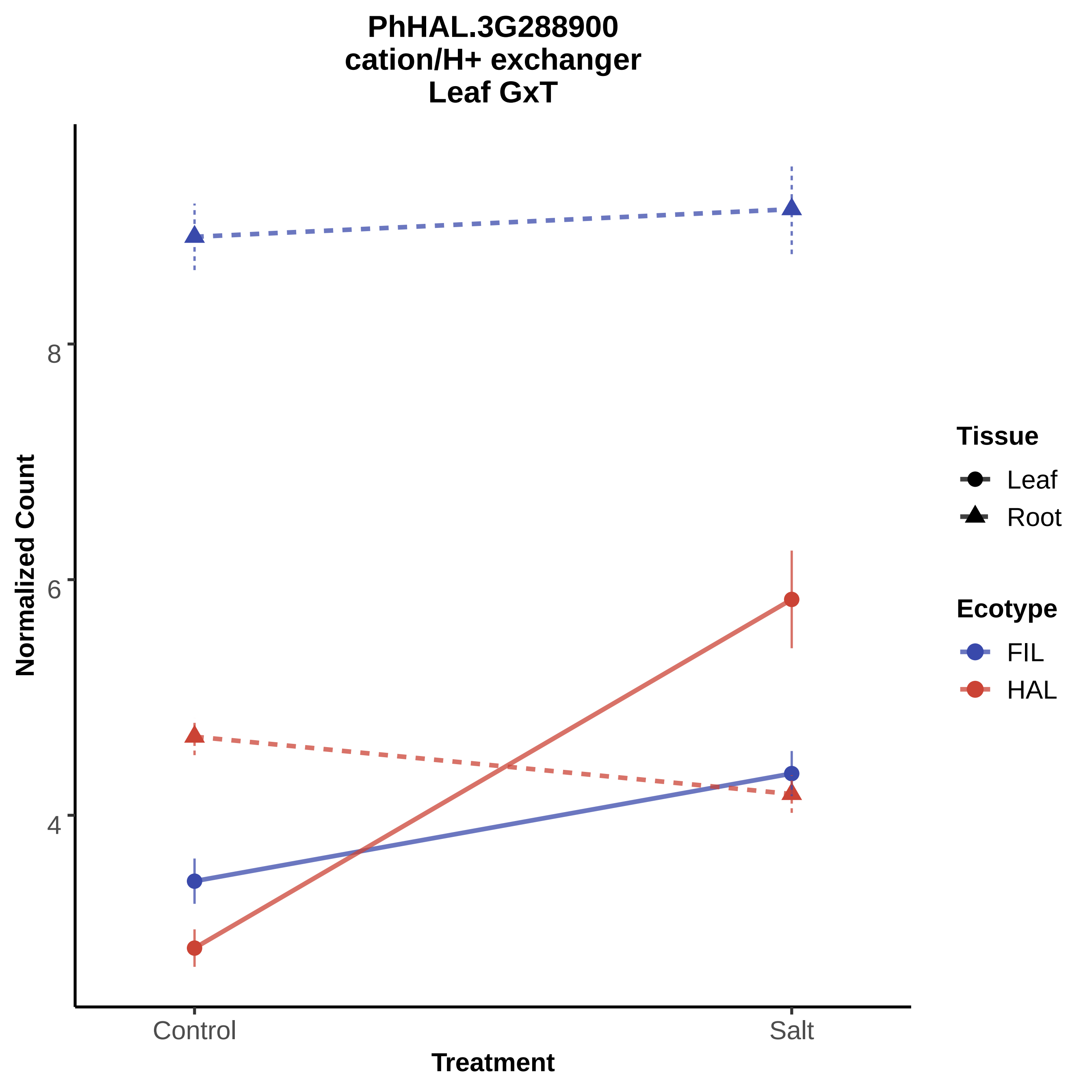
PhHAL.3G288900
cation/H+ exchanger
Leaf GxT
Normalized Count
8
6
4
Control
Salt
Treatment
Tissue
Leaf
Root
Ecotype
FIL
HAL

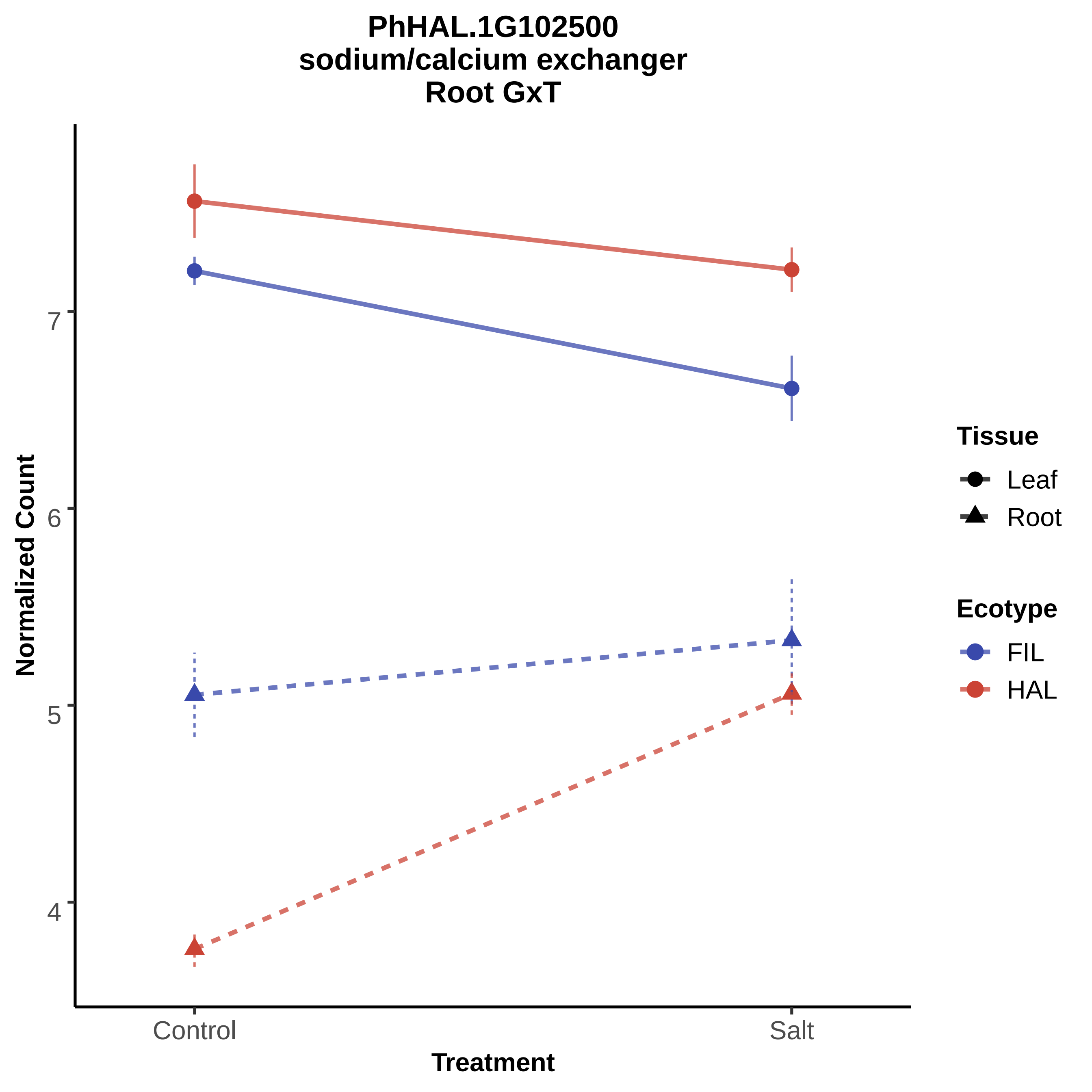

PhHAL.1G102500
sodium/calcium exchanger
Root GxT
Normalized Count
7
6
5
4
Control
Salt
Treatment
Tissue
Leaf
Root
Ecotype
FIL
HAL

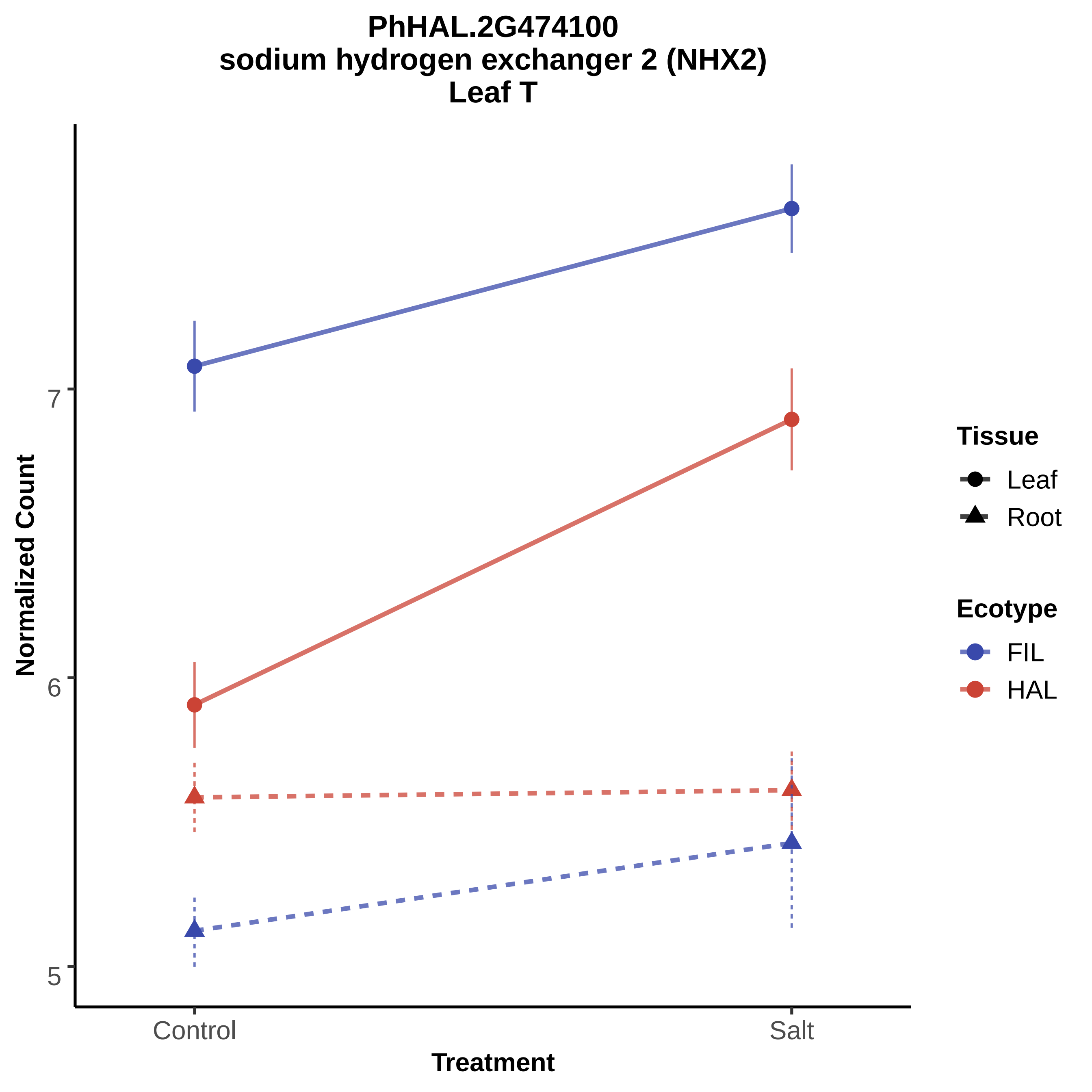
PhHAL.2G474100
sodium hydrogen exchanger 2 (NHX2)
Leaf T
Normalized Count
7
6
5
Control
Salt
Treatment
Tissue
Leaf
Root
Ecotype
FIL
HAL

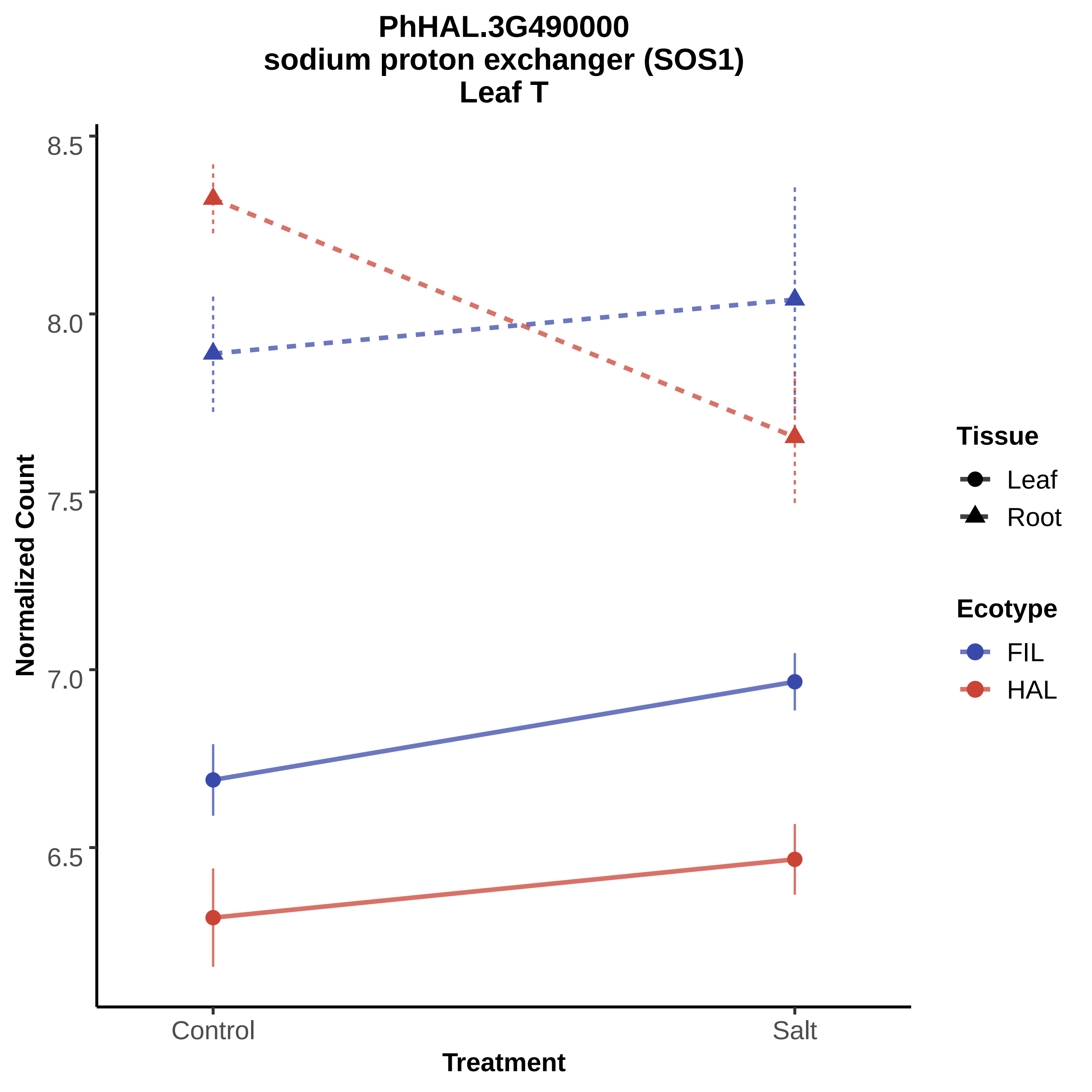
PhHAL.3G490000
sodium proton exchanger (SOS1)
Leaf T
Normalized Count
8.5
8.0
7.5
7.0
6.5
Control
Salt
Treatment
Tissue
Leaf
Root
Ecotype
FIL
HAL

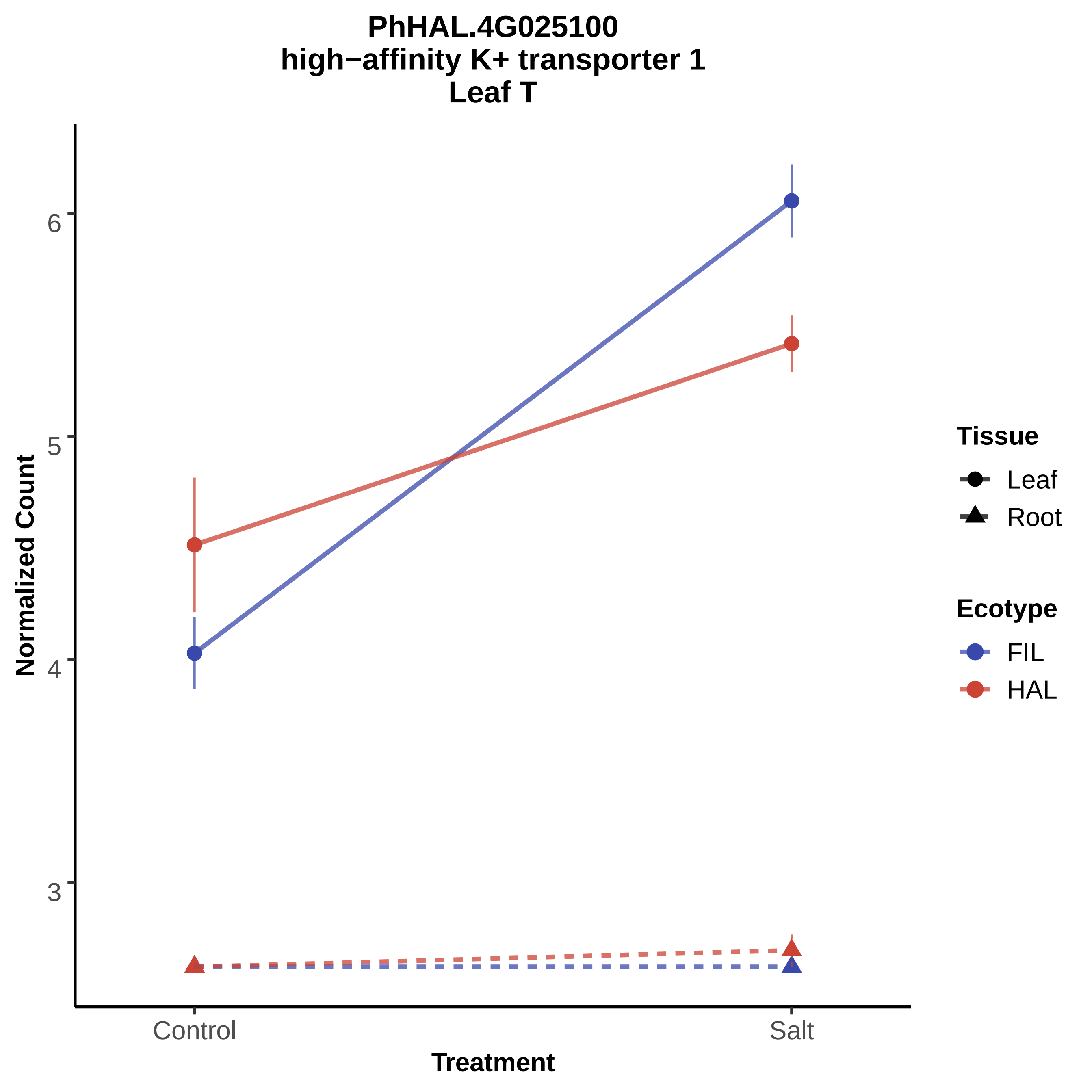
PhHAL.4G025100
high−affinity K+ transporter 1
Leaf T
Normalized Count
6
5
4
3
Control
Salt
Treatment
Tissue
Leaf
Root
Ecotype
FIL
HAL

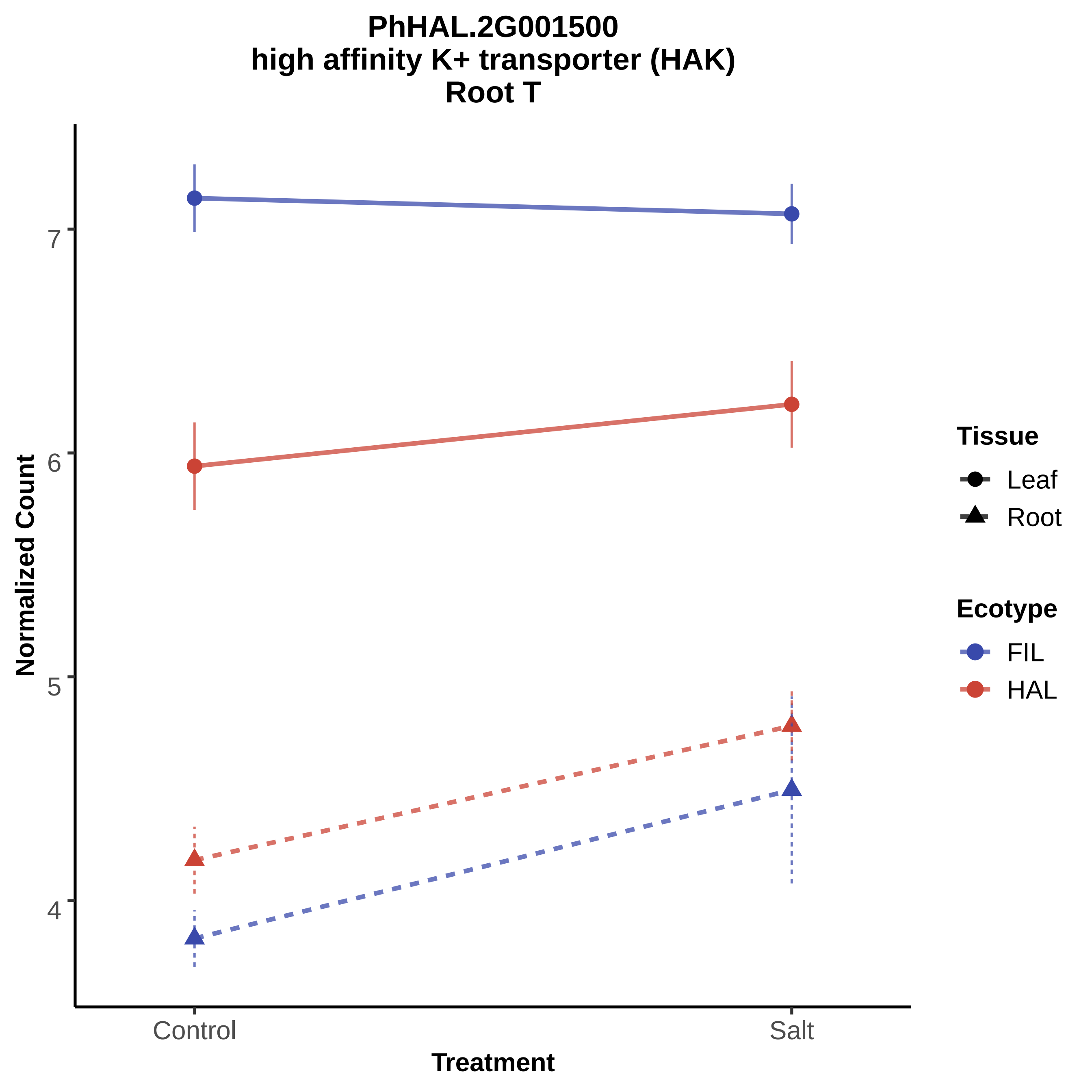

PhHAL.2G001500
high affinity K+ transporter (HAK)
Root T
Normalized Count
7
6
5
4
Control
Salt
Treatment
Tissue
Leaf
Root
Ecotype
FIL
HAL

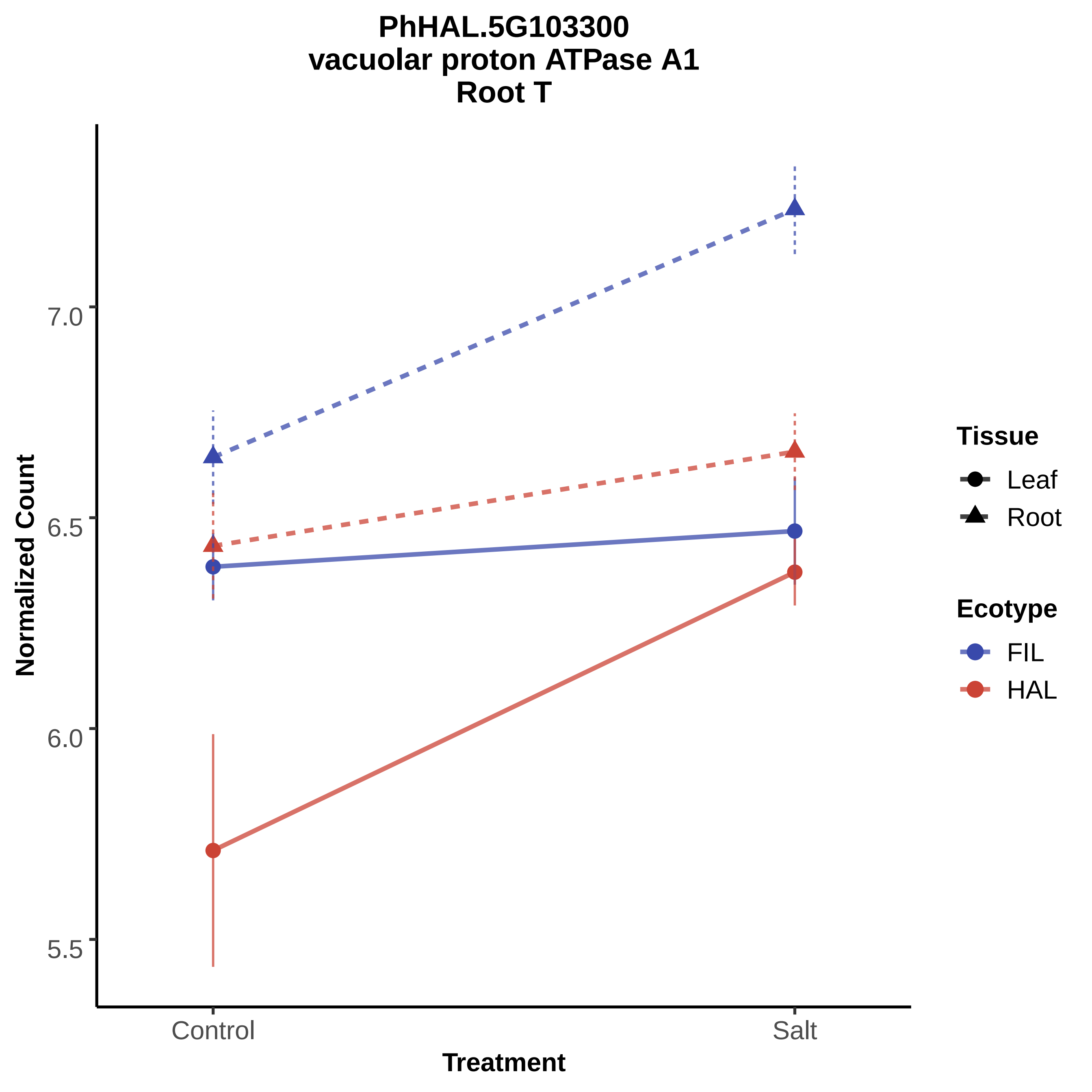

PhHAL.5G103300
vacuolar proton ATPase A1
Root T
Normalized Count
7.0
6.5
6.0
5.5
Control
Salt
Treatment
Tissue
Leaf
Root
Ecotype
FIL
HAL
